# Data-driven enterosignatures link gut microbiome reorganization to heat stress responses in lactating sows

**DOI:** 10.3389/fmicb.2026.1797687

**Published:** 2026-04-10

**Authors:** Chiara Gini, Francesco Tiezzi, Jicai Jiang, MaryKate H. Byrd, Hui Wen, Jay S. Johnson, Luiz F. Brito, Stephan van Vliet, Christian Maltecca

**Affiliations:** 1 Department of Animal Science, North Carolina State University, Raleigh, NC, United States; 2 Department of Agriculture, Food, Environment, and Forestry, University of Florence, Firenze, Italy; 3 Department of Animal Sciences, Purdue University, West Lafayette, IN, United States; 4 Division of Animal Sciences, University of Missouri, Columbia, MO, United States; 5 Department of Nutrition, Dietetics, and Food Sciences, Center for Human Nutrition Studies, Utah State University, Logan, UT, United States

**Keywords:** enterosignature, genetic background, heat stress, lactating sows, metagenomics, NMF

## Abstract

**Background:**

Heat stress (HS) can disrupt the gut microbiome, yet most livestock studies rely on taxonomic summaries that overlook the ecological structure of microbial communities. Enterosignatures (ES) as latent, co-occurring microbial assemblages learned from metagenomic data, offer a framework to capture these dynamics but have scarcely been applied in livestock HS research.

**Methods:**

Shotgun metagenomes were obtained from 25 lactating sows, belonging to two genetic lines (TOL, *n* = 13; SEN, *n* = 12), which were divergently selected based on genomic breeding values (GEBVs) for heat tolerance, and exposed to HS conditions. Results were decomposed using non-negative matrix factorization (NMF), yielding 8 taxonomic (T-ES) and 5 functional (F-ES) subcommunities. Functional profiles (based on KEGG Orthology, KOs) were mapped to metagenome-assembled genomes (MAGs) to integrate metabolic attributes within each ES.

**Results:**

Temporal shifts dominated T-ES variation, with limited genetic-line effects. T-ES 1 (*p* = 5.42 × 10^−4^, Cohen’s *d* = 0.723) and T-ES 7 (*p* = 0.007, Cohen’s *d* = 0.303) showed increases from day 4 to day 14. Despite modest overall genetic line effects, TOL animals progressively transitioned toward phylogenetically diverse and balanced communities, whereas SEN animals shifted toward imbalanced states characterized by enrichment of taxa with pathobiont potential or single-taxon dominance. Other T-ES displayed small to moderate effects, and T-ES 8 showed a potentially noteworthy genetic line-specific effect size at late lactation (Cohen’s *d* = 0.960; 95% CI: −1.80 to −0.10), though omnibus tests were non-significant (*p* = 0.757), and the wide confidence interval underscores substantial uncertainty at this sample size. No F-ES reached statistical significance (*p* > 0.05); moderate effect sizes (up to *d* = 0.638) suggest possible functional restructuring warranting investigation in larger cohorts.

**Conclusion:**

This work presents the first use of ES to track microbiome responses to HS in lactating sows. ES revealed latent taxonomic and functional subcommunities with clear temporal reorganization, offering insights not detectable with standard clustering or diversity metrics. Although genetic-line effects were modest, several ES showed biologically relevant shifts, supporting ES as a hypothesis-generating exploratory framework for linking microbial ecology to physiological adaptation under HS conditions, while warranting validation in larger, controlled trials.

## 1 Introduction

The gut microbiome is a highly complex, dynamic ecosystem that is closely linked to host physiology and health. Various frameworks have been developed to describe its structure, each targeting a different level of organization. Early work focused mainly on taxonomy, summarizing communities by phylum or genus and using simple metrics such as the Bacteroidota:Firmicutes ratio (Ley et al., 2006). More recent approaches have moved beyond these simplistic measures toward functional guild-based frameworks (Frioux et al., 2023). These taxonomic summaries yielded important initial insights but offered limited ecological context. For example, they could not distinguish taxa that simply co-occur from those that actively interact or occupy similar niches. To capture this missing ecological dimension, the concept of enterotypes (ET) was introduced (Wu et al., 2011). ET group genus-level profiles into discrete community types, typically using algorithms such as partitioning around medoids or Dirichlet multinomial mixture models (Arumugam et al., 2011; Wu et al., 2011; Holmes et al., 2012). Although widely used, these methods enforce a categorical view in which each sample is assigned to a single dominant type (Costea et al., 2018), even though gut communities often represent mixtures of multiple assemblages. As an alternative, microbial guilds group taxa based on shared ecological or metabolic functions rather than strict taxonomic relatedness. Microbial guilds offer an alternative to the exclusive classification of the ET by grouping taxa by shared ecological or metabolic functions rather than strict taxonomy. Guild-based analyses help reveal cooperation, complementarity, and functional redundancy within the microbiome, shedding light on how specific groups of microbes sustain similar ecological roles across hosts or environments (Frioux et al., 2023). However, these guilds are typically defined *a priori*, relying on pre-existing biological knowledge, which can limit their ability to uncover unexpected or context-specific associations.

To move beyond these limitations, enterosignatures (ES) have recently been proposed and provide a data-driven framework for identifying latent microbial subcommunities through matrix factorization or topic-modeling approaches (Frioux et al., 2023; Vourlaki et al., 2025). Each ES can describe a cluster of Metagenome-Assembled Genomes (MAGs) that co-occur across samples, and each sample can express a combination of these clusters to different extents. Thus, individual microbiomes are no longer forced into discrete types but are instead described as a combination of underlying ecological signals. Approaches such as non-negative matrix factorization (NMF) (Breuninger et al., 2021) or latent Dirichlet allocation (Cai et al., 2017; Hosoda et al., 2020) make this possible by learning latent variables that capture the proportional contribution of each microbial assemblage. NMF decomposes the abundance matrix into two non-negative matrices: one describing latent microbial subcommunities and another describing how strongly each sample expresses those subcommunities. The non-negative constraint preserves the additive nature of microbial abundances and ensures that resulting components are biologically interpretable (Lee and Seung, 1999; Cai et al., 2017). Compared with ET or predefined guilds, ES preserve within-sample heterogeneity and provide a flexible, mechanistic lens on microbial community structure. Despite their conceptual advantages, ES are relatively new and have been seldom applied in agricultural animal systems, leaving their potential for elucidating environmental stress responses largely unexplored.

Lactating sows represent a particularly promising animal system in which to study gut microbiome dynamics. Exposure to heat stress (HS) during lactation is known to profoundly influence feed intake, milk production, and future reproductive performance, among other production and welfare parameters, while also inducing metabolic and physiological changes that affect gut function and microbial composition (He et al., 2019a; Le Sciellour et al., 2019; Xiong et al., 2020). These physiological disruptions are typically studied in relation to inflammatory responses, shifts in energy allocation, alterations in gut permeability, and changes in nutrient absorption, all of which may reshape microbial communities (Mayorga et al., 2019). These alterations may propagate to offspring (Johnson and Baumgard, 2019) and impact overall herd productivity, emphasizing the need to understand how environmental stressors reshape the gut ecosystem. However, most studies to date have relied on taxonomic summaries or global diversity metrics, which provide limited insight into how specific microbial assemblages reorganize under HS conditions and how these subcommunities relate to host traits (He et al., 2019b; Jeong et al., 2021; Xia et al., 2022). Given the dynamic and multifactorial nature of the sow gut microbiome under HS, ES could offer a particularly powerful approach. In fact, by identifying latent, co-occurring microbial communities and quantifying their relative contributions across treatments and/or time, ES enables the identification of latent subcommunities that can be statistically associated with physiological responses or production traits, thereby providing a framework for hypothesis generation regarding microbiome-targeted strategies (Frioux et al., 2023; Vourlaki et al., 2025). This framework could offer a nuanced view of microbiome–host interactions otherwise difficult to achieve with discrete clustering alone, particularly in longitudinal contexts where microbial community composition evolves continuously and multiple ecological processes act simultaneously.

This study applied NMF-based ES decomposition to gut metagenomes from lactating sows belonging to two divergent genetic lines (heat-tolerant, TOL, and heat-sensitive, SEN; divergently selected by their genomic breeding values for heat-tolerance; Byrd et al., 2025; Freitas et al., 2023) under HS conditions with three objectives: (1) derive data-driven taxonomic and functional subcommunities from MAGs and KEGG Orthology identifiers (KO) data; (2) characterize ES temporal dynamics across lactation; and (3) explore associations between ES profiles and host physiology to generate hypotheses about microbiome–host interactions during thermal challenge. NMF was computed across candidate component numbers, with k (where k is the optimal number of ES) selected based on stability and reconstruction metrics. KO annotations were mapped to MAGs to enable genome-resolved functional characterization. Given the modest sample size and observational design, in which genetic line differences cannot be fully disentangled from other sources of individual variation, effect sizes were reported alongside significance values to aid interpretation.

## 2 Materials and methods

### 2.1 Study design and data collection

All data analyzed in this study were derived from biological samples and phenotypic measurements previously collected by Byrd et al. (2025), whose animal procedures were approved by the Purdue University Animal Care and Use Committee (protocol #2101002105). Since our work relied entirely on these existing datasets and did not involve additional animal handling, no new ethical approval was required. Briefly, the study employed 25 multiparous sows (parities 2–5) selected from a Duroc × (Landrace × Yorkshire) cohort previously evaluated basing on Genomic Estimated Breeding Values (GEBV) for heat resilience utilizing the phenotyping protocols and vaginal temperature assessment methods as described previously (Wen et al., 2023; Usala et al., 2021). The most heat-tolerant (TOL, *n* = 13) and heat-sensitive (SEN, *n* = 12) animals were transferred to the USDA-ARS Livestock Behavior Research Unit for individual housing. Sows were subjected to a diurnal HS challenge from farrowing to piglet weaning. This challenge involved cyclic temperature fluctuations—32°C during daylight hours (0800–1,700 h) and 28°C overnight (1,700–0800 h)—with relative humidity regulated between 50 and 70% to simulate a natural environment.

#### 2.1.1 Metagenomic data

Fecal samples for metagenomic profiling were collected from the above described TOL and SEN sows on day 4, 8, and 14 of lactation, choosing these days as representatives timepoints for the microbiome composition during early-, mid-, and late-HS, respectively. Samples were transferred into sterile tubes, stored at −80°C, and processed to obtain a total of 75 samples sequenced. Nucleic acids were extracted using the PowerFecal DNA/RNA Purification Kit (QIAGEN^®^). DNA quality and quantity were evaluated with Qubit (Thermo Fisher^®^) and TapeStation (Agilent^®^). Libraries were prepared using the ThruPLEX DNA Library Prep Kit (Takara^®^), pooled equimolarly, and sequenced on an Illumina NovaSeq 6000 (150 bp paired-end). Raw reads were for with the ATLAS workflow (v2.18.2) (Kieser et al., 2020), including quality control, assembly with metaSPAdes (Nurk et al., 2017), gene prediction, and functional and taxonomic annotation using DRAM (Shaffer et al., 2020), GTDB-Tk (Chaumeil et al., 2020), and KO databases using default parameters (Kanehisa and Goto, 2000). MAGs were dereplicated within ATLAS at 99% average nucleotide identity (ANI), corresponding to approximately strain-level resolution, with taxonomic assignment performed by GTDB-Tk at species-level thresholds (95% ANI).

#### 2.1.2 Phenotypic data

Phenotypic measurements were also obtained from the above-mentioned animals. Extending beyond the approach by Byrd et al. (2025), the present analysis focused on only the traits corresponding to, or traceable to, the metagenomic sampling days. To harmonize temporal resolution, lactation days were grouped into three intervals (0–4, 5–8, and 9–14 lactation days), and phenotypic values within each interval were averaged. The integrated phenotypic dataset included respiration rate (RR; acts per minute; 0800, 1,200, 1,600, and 2,000 h daily), skin surface temperatures (ear, shoulder, rump, and tail; °C; 0800, 1,200, 1,600, and 2,000 h daily), daily feed intake (kg/day) and water intake (L/day), and additional variables measured at specific time points such as automatically-monitored vaginal temperature (°C; 15 min intervals), sow and litter body weight (on sampling days), salivary cortisol (ELISA; the day before sampling), respiratory quotient (RQ; on sampling days; CO_2_/O_2_), and total heat production (THP; on sampling days; kcal/h kg body weight^0.75^) (Supplementary Table 1). Details on animal selection, housing, lactation management, and phenotypic measurements can be found in Byrd et al. (2025).

### 2.2 Non-parametric functional and taxonomic clustering

#### 2.2.1 Matrices preparation and filtering

From metagenomic sequencing, a matrix of MAG abundances (samples × MAGs) was generated, and a corresponding KO abundance matrix (samples × KO) was constructed by aggregating gene-level KO annotations weighted by the abundances of their respective MAGs. For both data types, features (MAG or KO) were retained if they were present in ≥ 20% of samples or had a mean abundance above the 75th percentile, balancing sparsity reduction with preservation of biological signal. All data processing and filtering steps were performed using the tidyverse (v2.0.0) R package (Wickham et al., 2019). To visualize overall community structure and genetic line-level variation prior to ES decomposition, dimensionality reduction was performed on the MAG abundance matrix. Principal Component Analysis (PCA) was applied to the log-transformed, scaled matrix using FactoMineR (v2.11) (Lê et al., 2008), to capture linear variation, while UMAP (Uniform Manifold Approximation and Projection) (Healy and McInnes, 2024) was applied to the same matrix using cosine distance with n_neighbors = 5 and min_dist = 0.1 (UMAP R package v0.2.10.0) (Healy and McInnes, 2024) to preserve local neighborhood structure. These complementary approaches provided an initial assessment of sample separation by timepoint and genetic line before proceeding to NMF-based subcommunity identification. Ecological niche space was visualized in PCA space using convex hulls and 95% confidence ellipses around group centroids, with temporal trajectories connecting timepoint-specific centroids. Visualizations were created with ggplot2 (v3.4.4) (Wickham, 2016), patchwork (v1.2.0) (Pedersen, 2025), and ComplexHeatmap (v2.16.0) (Gu et al., 2016).

#### 2.2.2 Non-negative matrix factorization

**FIGURE 1:**
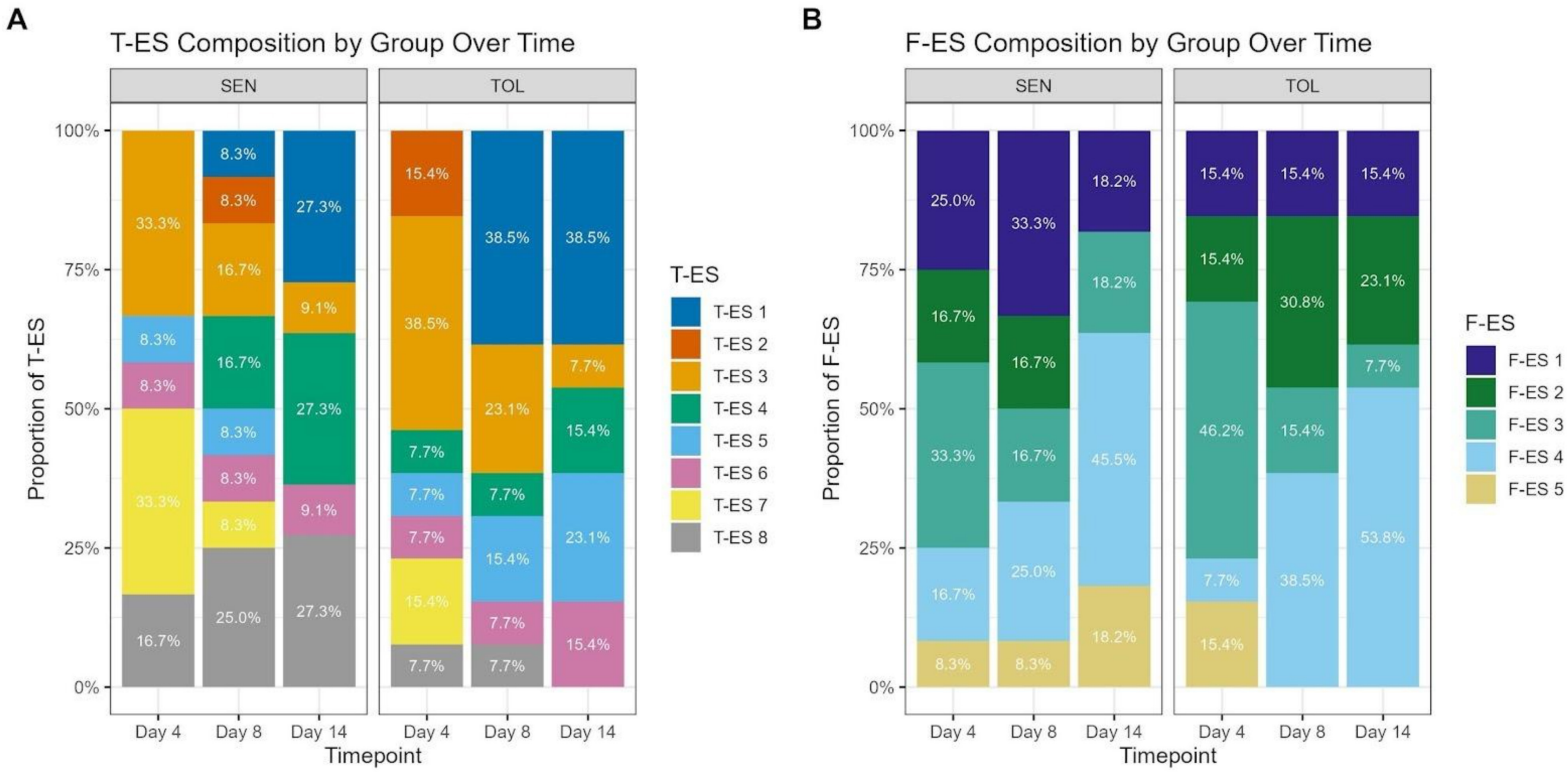
Temporal and genetic line patterns of ES. **(A)** Relative composition of T-ES factors at the MAG level across genetic lines and timepoints. Bars are stacked to show the contribution of each T-ES (T-ES 1–8), with percentages labeled within the bars. **(B)** Relative composition of F-ES factors at the KO level across the same genetic lines and timepoints. Bars are stacked to show the contribution of each F-ES (F-ES 1–5), with percentages labeled within the bars.

NMF was performed using the Brunet algorithm (Brunet et al., 2004) implemented in the NMF R package (v0.27) (Gaujoux and Seoighe, 2010). Input matrices for MAG and KO were normalized by total sum scaling (TSS), which converts raw abundances to within-sample proportions. TSS is the standard preprocessing for NMF-based microbiome decomposition because it satisfies the non-negativity constraint required by NMF while removing library-size effects (Frioux et al., 2023; Vourlaki et al., 2025). Rarefaction was not performed, as TSS achieves comparable library-size normalization without the information loss inherent in random subsampling (McMurdie and Holmes, 2013). Optimal factorization rank (number of ES) was determined using a consensus tolerance-based approach evaluating five quality metrics: Cophenetic correlation, silhouette width (Rousseeuw, 1987), residual sum of squares, dispersion, and explained variance. For each metric, ranks within 10% (taxonomy analyses) or 5% (functional analyses) of the optimal value were deemed acceptable. The rank accepted by the most metrics was selected. This consensus-based rank selection represents a pragmatic approach given the exploratory nature of this study; however, alternative methods such as cross-validation or Bayesian model comparison could yield different optimal ranks. Sensitivity analyses examining adjacent ranks (*k* ± 1) showed qualitatively similar ES compositions, suggesting the biological interpretation is robust to minor rank variation, though we acknowledge this remains a methodological limitation inherent to unsupervised factorization. Final NMF solutions used 30 independent runs with the optimal rank to ensure convergence, using a fixed random seed (12,345) for reproducibility. The NMF decomposition generated a basis matrix W (samples × ES), representing the loading of each ES across samples, and a coefficient matrix H (ES × features, with features corresponding to MAGs in the T-ES analysis and KO in the F-ES analysis). Features were ranked within each ES using z-score standardization when enrichment showed low variability (coefficient of variation < 0.5), and by fold-enrichment relative to the mean loading across ES otherwise. For descriptive visualization (e.g., Figure 1), each sample was assigned to its dominant ES defined as the factor with the highest loading in the W matrix (argmax). This discrete assignment was used solely for illustrative purposes; all statistical analyses used the continuous loading values from the W matrix as response variables, preserving the mixed-membership nature of the NMF decomposition. For taxonomic analyses, T-ES were defined as the top 30 MAGs per factor, annotated with phylum, class, order, family, and genus classifications when available. For functional analyses, F-ES were defined as the top 30 KO per factor ranked by z-score (i.e., standardized abundance relative to the mean across samples) or enrichment level. KO functional categories were assigned by keyword matching to KEGG annotations (Kanehisa and Goto, 2000), grouping functions into more than 20 categories including transport, kinases, metabolism, biosynthesis, and others.

#### 2.2.3 NMF stability validation

Subsampling stability analysis (100 iterations, 80% random subsamples with Hungarian algorithm matching) was performed to evaluate the robustness of the selected factorization ranks. In each iteration, NMF was refitted at the established ranks, and top-feature membership was compared to the reference solution using Jaccard similarity with optimal bipartite matching (Hungarian algorithm). Feature persistence across subsamples was additionally quantified as the proportion of top-ranked features recovered in ≥ 80% of iterations. This procedure allowed assessment of signature reproducibility independently of the original full-sample solution.

#### 2.2.4 Linear mixed models

Both T-ES and F-ES were analyzed using linear mixed-effects models. ES loadings from the W matrix were normalized in a two-step procedure for downstream statistical testing. First, trimmed mean of M-values (TMM) normalization was applied to the loading matrix to account for composition bias across samples (i.e., differences in total ES loading that may arise from varying microbial biomass or sequencing depth). Second, centered log-ratio (CLR) transformation was applied to address the compositional constraint inherent in NMF loadings, where proportional contributions across ES sum to a constant within each sample. Importantly, TMM and CLR were applied to the NMF-derived loading matrix, not to the raw abundance data: the raw abundances were normalized by TSS prior to NMF (see above), while this second normalization stage addresses different biases in the derived latent factor space. For all models, genetic lines (TOL and SEN) and time point (day 4, day 8, and day 14) were included as categorical fixed effects with interaction, while animal ID was included as a random intercept to account for repeated measurements within the same animal to ensure proper partitioning of within- and between-animal variance. To characterize ES dynamics, the response variable was the normalized loading of each ES, modeled as:


$$y_{i\,j\,k}=\mu +\tau_{i}+\gamma_{j}+{\left(\tau \,\gamma \right)}_{i\,j}+a_{k}+\varepsilon_{i\,j\,k}$$

where *y*_*ijk*_ is the CLR-transformed ES load for time point *i*, genetic line *j*, and animal *k*; μ is the overall mean; τ_*i*_ is the fixed effect of time point (*i* = day 4, 8, 14); γ_*j*_ is the fixed effect of genetic line (*j* = TOL, SEN); (*τγ*)_*ij*_ is their interaction; $a_{k}∼N\,\left(0,\sigma_{a}^{2}\right)$ is the random intercept for animal; and $\varepsilon_{i\,j\,k}∼N\,\left(0,\sigma_{e}^{2}\right)$is the residual error. For microbiome–host associations, each physiological phenotype was modeled as a function of ES load, time point, and genetic line:


$$P_{i\,j\,k\,l}=\mu +\beta \cdot E\,S_{i\,j\,k\,l}+\tau_{i}+\gamma_{j}+{\left(\beta \,\tau \right)}_{i\,l}+{\left(\beta \,\gamma \right)}_{j\,l}+$$

$${\left(\tau \,\gamma \right)}_{i\,j}+{\left(\beta \,\tau \,\gamma \right)}_{i\,j\,l}+a_{k}+\varepsilon_{i\,j\,k}$$

where *P*_*ijkl*_ is the phenotype value (including RR, skin surface temperatures, daily feed intake, water intake, vaginal temperature, sow and litter body weight, salivary cortisol, RQ, THP); *ES*_*ijkl*_ is the ES load (continuous covariate); β is its regression coefficient; and remaining terms denote fixed effects, their two- and three-way interactions, a random animal intercept, and residual error as above. In both models, type III ANOVA tables were extracted using Satterthwaite’s method for degrees of freedom approximation (Armstrong et al., 2022), with *p*-values adjusted using the false discovery rate (FDR) method (Benjamini and Hochberg, 1995).

##### 2.2.4.1 Model diagnostics

All linear mixed-effects models were inspected for convergence, residual normality (Q-Q plots), homoscedasticity (residual-versus-fitted plots), and random effect singularity. Models failing to converge were excluded from inference. The full model including all two- and three-way interactions was retained as a pre-specified analysis plan to avoid biasing the analysis toward main effects; however, we acknowledge that the inclusion of higher-order interactions with 25 animals carries a risk of overparameterization and interpret higher-order interactions cautiously.

#### 2.2.5 *Post-hoc* comparisons

Estimated marginal means (EMMs) were computed using emmeans (v1.10.0) (Lenth, 2023) for genetic line × time point combinations. Pairwise contrasts tested on the first model first the genetic line differences at each time point, and then the temporal changes within each genetic line. In the second model, contrasts were used to evaluate how phenotypic responses differed across ES loads, testing both genetic line-specific effects of ES at each time point and time-specific effects of ES within each genetic line. *P*-values were FDR-adjusted within each contrast family (i.e., within each phenotype × ES combination) using the multcomp R package (v1.4-25) (Hothorn et al., 2008). This per-family correction is less conservative than global correction across all phenotype–ES combinations; the total testing burden across 16 phenotypes and multiple ES should be considered when interpreting results.

#### 2.2.6 Effect sizes

Given the exploratory nature of this framework and the modest sample size, effect sizes were interpreted in conjunction with corresponding *p*-values and 95% confidence intervals to capture biologically meaningful patterns warranting validation in larger cohorts. Effect sizes were calculated as Cohen’s *d* (Cohen, 1988) with 95% confidence intervals using the effectsize package (v0.8.6) (Ben-Shachar et al., 2020). Cohen’s d was computed as the difference between genetic line means divided by the pooled standard deviation, with interpretation following conventional thresholds: small (0.2 < | *d*| < 0.5), medium (0.5 < | *d*| < 0.8), and large (| *d*| > 0.8) effects (Cohen, 1988).

#### 2.2.7 Diversity and niche metrics

Taxonomic and functional niche breadth was quantified using Shannon diversity (H’) (Shannon, 1948) of ES loadings per sample:


$$H^{\prime }=-\Sigma \left(p_{i}=ln\left(p_{i}\right)\right)$$

where *p*_*i*_ is the proportion of loading in factor *i*. Evenness was calculated as H’/log(nfactors) (Pielou, 1966). Models were inspected for convergence warnings and singularity. Factors failing to converge were excluded from statistical inference. Statistical significance was assessed at α = 0.05 after FDR correction, with marginal significance noted at α = 0.10. Large effect sizes were defined as | Cohen’s *d*| > 0.8 (Cohen, 1988).

#### 2.2.8 Correlation analysis between T-ES and F-ES

To investigate the relationship between T-ES and F-ES, the sample score matrices derived from NMF decomposition of T-ES (*k* = 8) and F-ES (*k* = 5) data were compared across all 74 samples. Pairwise Spearman rank correlations were computed between all T-ES and F-ES score vectors, yielding a 8 × 5 correlation matrix. Statistical significance was assessed for each of the 40 pairs and *p*-values were retained for annotation.

#### 2.2.9 Software and visualization

All analyses were performed in R version 4.3.1 (R Core Team, 2020). Additional packages included viridis (v0.6.4) for color palettes, pheatmap (v1.0.12) for heatmaps, circlize (v0.4.16) (Gu et al., 2014) for ComplexHeatmap color mapping, ggrepel (v0.9.5) for label positioning, networkD3 (v0.4) for interactive network visualizations, and Hmisc and ggplot2 for the computation and visualization of Spearman rank correlations between T-ES and F-ES. Statistical significance annotations followed conventional notation: ***P < 0.001, **P < 0.01, *P < 0.05, ⋅*P* < 0.10.

## 3 Results

### 3.1 Sample composition and availability

A total of 75 fecal metagenomes from 25 sows at 3 lactation time points (days 4, 8, and 14) were obtained, and 74 were included in the analyses (1 sample was discarded due to poor sequencing quality). Sequencing yielded a mean of 79.6 ± 15.1 million raw read-pairs per sample (range: 0.34–109 M), with 45.9 ± 25.4 million read-pairs retained after quality control. Detailed per-sample sequencing and assembly statistics are provided in Supplementary Table 1. Assembly and binning via Metagenome-ATLAS recovered a non-redundant set of 835 MAGs dereplicated at a 95% ANI threshold across all samples (mean N50: 39.0 kb; mean completeness: 89.5 ± 10.8%; mean contamination: 0.66 ± 0.87%), of which 60.6% were classified as high-quality following MIMAG standards (Bowers et al., 2017). The mentioned sample size was determined by the availability of animals from the parent study (Byrd et al., 2025) that were pre-characterized for genetic heat tolerance. While this sample size provides sufficient power (>80%) to detect large effects (Cohen’s *d* ≥ 0.8) with α = 0.05 in between-genetic line comparisons, power to detect medium effects (*d* = 0.5) is approximately 50%, and small effects (*d* = 0.2) would require substantially larger cohorts. This power limitation, inherent to exploratory livestock microbiome studies, means that non-significant results should not be interpreted as absence of evidence. Effect sizes are reported alongside *p*-values to facilitate interpretation of biological relevance, support future meta-analytic synthesis, and inform power calculations for confirmatory studies. Throughout, we distinguish between statistically supported findings and exploratory signals identified by effect size magnitude alone.

### 3.2 Global community structure

**FIGURE 2:**
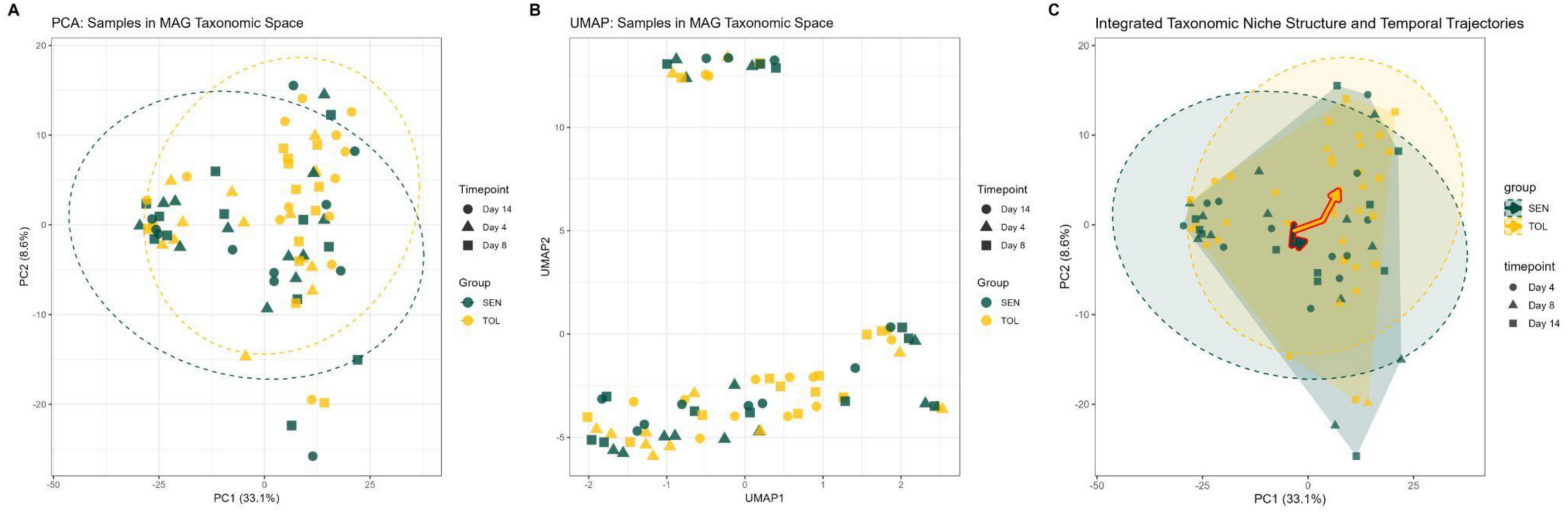
MAG Taxonomic structure across genetic lines and timepoints. **(A)** principal Component analysis (PCA) of MAG abundances, showing the separation of samples by genetic line (TOL vs. SEN) and timepoint, with 95% confidence ellipses indicating genetic line-specific variation. PC1 and PC2 explain 33.1 and 8.6% of the total variance, respectively. **(B)** UMAP dimensionality reduction, representing MAG compositional differences across samples in a nonlinear low-dimensional space, highlighting genetic line-specific clustering patterns over time. **(C)** Ecological niche representation, integrating PCA-derived positions to show group centroids, convex hulls, and temporal trajectories across days 4, 8, and 14. Trajectories indicate divergent paths of TOL and SEN genetic lines toward similar functional endpoints, with arrows illustrating directionality over time.

PCA of MAG abundance profiles revealed that the first principal component (PC1) explained 33.1% of the total variance, while PC2 and PC3 accounted for 8.6 and 6.4%, respectively. In the PCA space, TOL and SEN samples were partially separated along PC1, with TOL samples generally shifting from negative to positive values over time, whereas SEN samples remained near negative PC1 values. Projections involving PC3 did not reveal additional group- or time-dependent structure (data not shown). Temporal progression was also evident, as days 4, 8, and 14 samples occupied distinct regions along both PCs, forming trajectories in the taxonomic space (Figure 2A). UMAP dimension reduction largely recapitulated this pattern, showing comparable genetic line separation and temporal ordering, while capturing additional non-linear relationships in the data (Figure 2B). Ecological niche representation further highlighted these dynamics: convex hulls indicated partial overlap between TOL and SEN overall niches, but the trajectories of genetic line centroids demonstrated divergent temporal paths, with TOL moving steadily toward positive PC1 and PC2 values and SEN showing more constrained shifts. These results collectively suggest that TOL and SEN communities occupy overlapping but dynamically distinct regions of MAG taxonomic space, reflecting both genetic line-specific composition and temporal reorganization (Figure 2C).

### 3.3 Non-negative matrix factorization of taxonomic and functional profiles

To characterize microbial community structure and its dynamics under HS, NMF was applied separately to taxonomic composition derived from MAG abundances and to functional gene content represented by KO groups. For the taxonomic analysis, NMF was applied to the TSS MAG abundance matrix (835 MAGs across 74 samples, with a matrix sparsity of 38.6%). After filtering, the MAG matrix was reduced to 764 MAGs with 34% of sparsity. NMF was performed across candidate ranks 2–8, with 10 runs per rank to estimate model stability. Cophenetic correlation identified all ranks (*k* = 2–8) as acceptable, while silhouette width supported only *k* = 2 (Supplementary Figure 1A). No rank was supported by 4 or more metrics, although ranks 7 and 8 achieved the highest overall support (3 out of 5 metrics). Higher ranks were preferred among tied solutions to maximize ecological resolution, as lower ranks risk collapsing biologically distinct subcommunities into composite factors, and therefore for the MAG-based analysis, *k* = 8 was selected as the optimal rank based on consensus of 3 of 5 metrics. This rank was preferred over lower alternatives because collapsing to fewer factors risked merging biologically distinct subcommunities into composite factors, particularly the SEN-specific ecologically imbalanced signatures (T-ES 2, 3, 8) that would be subsumed at *k* ≤ 6. Subsampling validation confirmed that 6 of 8 taxonomic signatures were stably recovered across 100 random 80% subsamples (mean Jaccard = 0.67; Supplementary Figure 3). Consequently, the final model was refitted at *k* = 8 using 30 independent Brunet runs with a fixed random seed to ensure convergence. This decomposition yielded a W matrix (samples × T-ES) representing the contribution of each T-ES to individual samples and an H matrix (T-ES × MAGs) capturing the contribution of each MAG to the T-ES. For the functional analyses, KEGG Orthologs were mapped to their corresponding MAGs, generating 643,763 MAG–KO associations across 74 samples. The resulting KO abundance matrix contained 6,176 unique KO (74 × 6,176) with 7.4% sparsity. After filtering low-prevalence KO, 6,045 KO were retained (74 × 6,045), with a 5.6% sparsity. On this matrix, acceptable cophenetic correlations were observed for ranks 2–8, and acceptable silhouette widths for ranks 2–5, whereas no rank met all 5 nor 4 metrics simultaneously (Supplementary Figure 1B). Nevertheless, ranks 2–8 satisfied at least 3 metrics, indicating a broad plateau of tolerable solutions. Based on overall stability and consistency across metrics, *k* = 5 was selected for downstream functional analyses, yielding F-ES 1–5. Subsampling stability analysis further evaluated reproducibility of the functional ES. Four of five ES showed moderate-to-strong recovery across 100 random 80% subsamples (F-ES 2 = 0.69; F-ES 1 = 0.63; F-ES 5 = 0.50; F-ES 4 = 0.41), whereas F-ES 3 showed low recovery (Jaccard = 0.11), consistent with its low prevalence across samples (*n* = 3). Feature persistence analysis indicated that 49.3% of top-30 features were recovered in ≥ 80% of subsamples (Supplementary Figure 4). These results indicate generally stable functional structure, though F-ES 3 represents a comparatively unstable component and should be interpreted cautiously. The difference in yield of NMF decompositions between taxonomic and functional data (8 T-ES vs. 5 F-ES) may reflect the inherently greater resolving power of taxonomic composition for differentiating community types, whereas functional redundancy, as distinct taxa share similar metabolic repertoires, could contribute to functional variation being captured in fewer latent ES.

### 3.4 MAG analysis results

#### 3.4.1 T-ES composition

Eight T-ES derived from MAG data displayed distinct phylogenetic compositions encompassing diverse bacterial and archaeal lineage. Here, loading represents the quantitative contribution of each MAG to an ES, enrichment reflects its relative overrepresentation within that ES compared with others, and specificity denotes the extent to which a MAG is uniquely associated with a single ES. T-ES 1 consisted of 30 MAGs spanning 9 phyla, marked by broad phylogenetic breadth. *Bacteroidota* (7 MAGs; total loading—representing the sum of ES contributions from these taxa = 0.007) and *Bacillota_A* (12 MAGs; total loading = 0.005) dominated, with top contributors including *Coprovivens* MAG627 and *UBA3789* MAG745 (both enrichment = 8.000; specificity = 1.0). Additional high-enrichment taxa from *Fibrobacterota* and *Spirochaetota* highlighted the highly diverse nature of this community. T-ES 2 contained 30 MAGs from 8 phyla, strongly dominated by *Actinomycetota* (8 MAGs; total loading = 0.096). The ES was shaped by 3 enriched *Corynebacterium* MAGs (enrichment between 7.1 and 8.0), *Enterococcus_B faecium* MAG787 (enrichment = 6.923), and *Helicobacter_A rappini* MAG028 (enrichment = 5.686). Archaeal representation included *Methanobrevibacter_A* (enrichment = 1.875). Overall, the ES reflected a community enriched in opportunistic/pathobiont-associated taxa. T-ES 3 included 27 MAGs from 5 phyla but showed extreme skew: *Lactobacillus amylovorus* MAG021 alone contributed 88% of total loading (enrichment = 0.267). Remaining taxa—mainly Bacillota and Bacteroidota—showed modest contributions (total loading < 0.03 combined). This ES represented a low-diversity, single-taxon-dominated state. While *Lactobacillus* dominance is not inherently pathological in livestock systems, the extreme skew (88% of total loading from a single MAG) represents a substantial reduction in community diversity. T-ES 4 comprised 30 MAGs from 6 phyla, dominated by Bacillota_A (23 MAGs; total loading = 0.020) and *Cyanobacteriota* (2 MAGs; mean enrichment = 7.835). Highly enriched MAGs included *CAG-873* MAG624 and *Muribaculum* MAG625 (both enrichment ≈ 8.0). Several health-associated taxa such *as Faecalibacterium prausnitzii_I* (enrichment = 7.916) characterized this balanced anaerobic fermenter community. T-ES 5 consisted of 30 MAGs from 5 phyla, heavily enriched in *Bacillota_A* (22 MAGs; total loading = 0.043). This ES featured multiple highly enriched commensals—including *Christensenella minuta* MAG131 (enrichment = 7.999) and *Blautia_A* MAG624 (enrichment = 7.994)—and abundant fiber-degrading specialists such as *Ruminococcus flavefaciens* strains (enrichment = 7.5–7.7). These taxa suggested a community optimized for carbohydrate and fiber metabolism. T-ES 6 comprised 30 MAGs from 6 phyla, dominated by *Bacillota_A* (17 MAGs; total loading = 0.113) and *Bacteroidota* (8 MAGs; total loading = 0.085). Key defining members included *Porphyromonas* MAG489, *Prevotella* MAG213, and *RGIG5681* MAG060 (all enrichment = 8.0; specificity = 1.0). Additional enriched taxa such as *Peptoniphilus_C* and *Parvimonas* indicated a proteolytic, mucosa-associated community. T-ES 7 consisted of 30 MAGs from 5 phyla, with *Bacillota_A* strongly dominant (20 MAGs; total loading = 0.023). Top contributors included *CAG-177* MAG377 (enrichment = 8.0), *F23-B02* MAG406 (enrichment = 7.984), and *Roseburia_A porci* MAG379 (enrichment = 7.909). Multiple *Faecousia* species and diverse *Oscillospirales* suggested a butyrate-oriented fermentative assemblage. T-ES 8 included 30 MAGs from 8 phyla, enriched in *Actinomycetota* (6 MAGs; 0.083), *Bacteroidota* (7 MAGs; total loading = 0.062), and *Bacillota_A* (7 MAGs; total loading = 0.036). Defining MAGs included *Mobiluncus porci* MAG015 (loading 0.035), *Olsenella* (7.505), and *Slackia* (enrichment = 7.449). A cluster of *Campylobacterota* MAGs (enrichment between 5.4 and 6.4) and several mucosa-associated *Bacillota_C* taxa contributed to this heterogeneous, mixed-niche community (Supplementary Table 2).

#### 3.4.2 Temporal and genetic line patterns of T-ES

T-ES distribution from the MAG-based analyses exhibited distinct patterns across time points and genetic lines, with greater complexity than observed in the functional analyses (Figure 2A). On day 4, SEN animals showed co-dominant representation of T-ES 3 (33.3%) and T-ES 7 (33.3%), with T-ES 8 at 16.7%, T-ES 5 at 8.3%, and T-ES 6 at 8.3%. TOL animals demonstrated a different initial composition with T-ES 3 at 38.5%, T-ES 2 and T-ES 7 each at 15.4%, and T-ES 4, T-ES 5, T-ES 6, and T-ES 8 each at 7.7%. By day 8, substantial shifts occurred in both genetic lines. In SEN animals, T-ES 8 became most prevalent at 25.0% of samples, with T-ES 3 and T-ES 4 each at 16.7%, and T-ES 1, T-ES 2, T-ES 5, T-ES 6, and T-ES 7 each at 8.3%. In TOL animals, a dramatic shift occurred with T-ES 1 increasing to 38.5% of samples (from 0% on day 4), T-ES 3 decreasing to 23.1%, T-ES 5 at 15.4%, and T-ES 4, T-ES 6, and T-ES 8 each at 7.7%. On day 14, in SEN animals, 3 ES showed equal prevalence: T-ES 1, T-ES 4, and T-ES 8 each represented 27.3% of samples, with T-ES 3 at 9.1% and T-ES 6 at 9.1%. In TOL animals, T-ES 1 maintained its dominant position at 38.5%, T-ES 5 represented 23.1%, T-ES 4 and T-ES 6 each 15.4%, and T-ES 3 decreased further to 7.7% (Figure 1A).

#### 3.4.3 Statistical analyses

**FIGURE 3:**
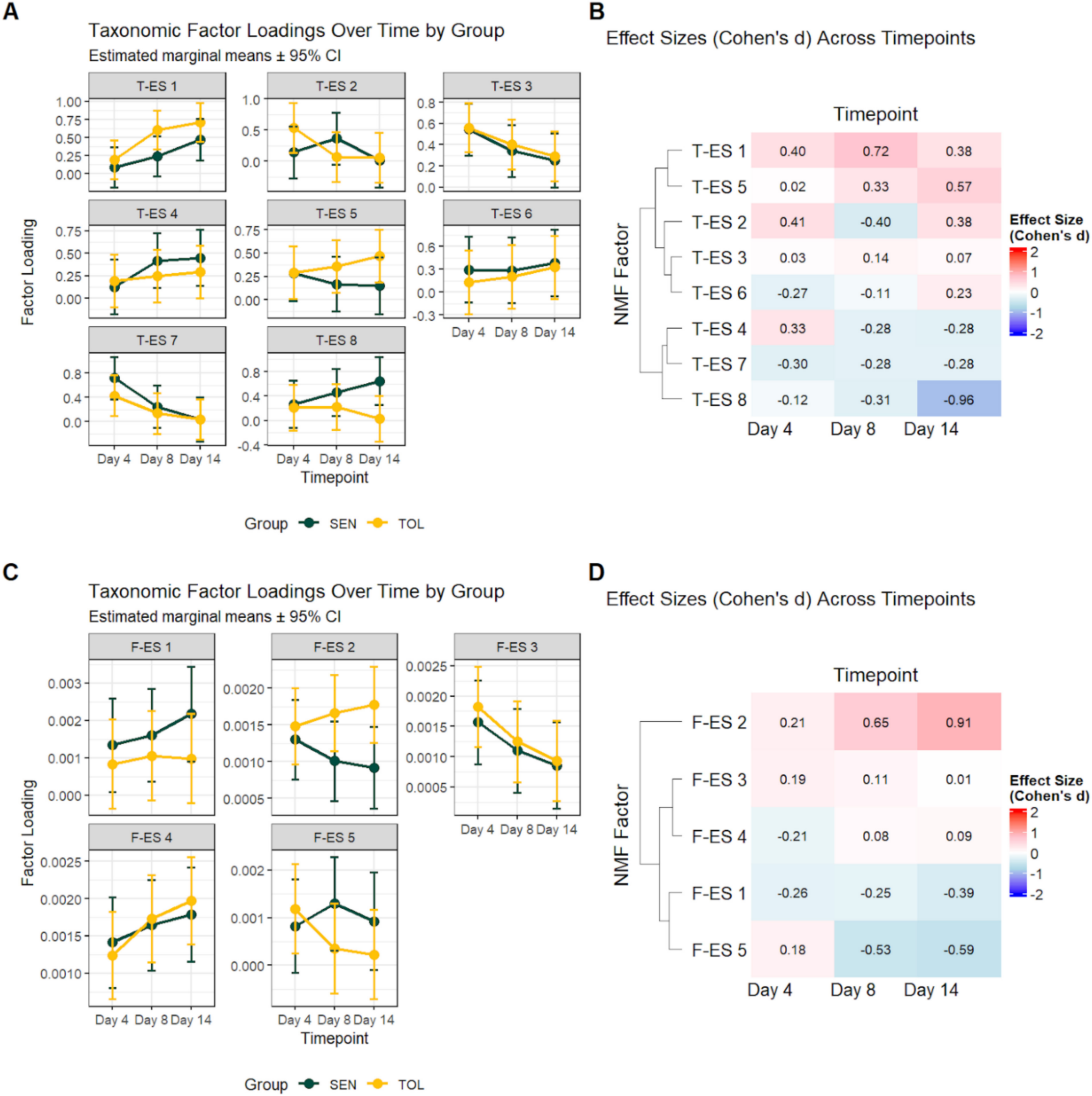
Temporal dynamics of NMF taxonomic ES and effect sizes across genetic lines. **(A)** Line plots of estimated marginal means ( ± 95% CI) of T-ES loadings across timepoints in TOL (blue) and SEN (red) animals. Each facet represents a different taxonomic T-ES 1–8, highlighting temporal trends and genetic line differences. **(B)** Heatmap of Cohen’s d effect sizes for each T-ES across timepoints, showing the magnitude and direction of changes between genetic lines. Positive values (red) indicate higher factor loadings in SEN animals, negative values (blue) indicate higher factor loadings in TOL animals, and values near zero (white) indicate minimal differences. Hierarchical clustering of rows reveals patterns of co-varying factors over time. **(C)** Line plots of estimated marginal means ( ± 95% CI) of F-ES loadings across timepoints in TOL (blue) and SEN (red) animals. Each facet represents a different functional F-ES 1–5, highlighting temporal trends and genetic line differences. **(D)** Heatmap of Cohen’s d effect sizes for each F-ES across timepoints, showing the magnitude and direction of changes between genetic lines. Positive values (red) indicate higher factor loadings in SEN animals, negative values (blue) indicate higher factor loadings in TOL animals, and values near zero (white) indicate minimal differences. Hierarchical clustering of rows reveals patterns of co-varying factors over time. ES, enterosignature; T-ES, taxonomic enterosignature; NMF, non-negative matrix factorization; TOL, thermotolerant; SEN, thermosensitive; CI, confidence interval.

Across the eight T-ES, linear mixed models showed limited evidence of genetic line- or interaction-driven differences, with temporal dynamics emerging as the primary driver of variability (Figures 3A,B). Specifically, T-ES 1 exhibited a highly significant time effect (*p* = 5.42 × 10^−4^), with genetic line effect *p* = 0.757, interaction *p* = 0.641, and maximum Cohen’s d = 0.723. Temporal contrasts indicated significant increases in both TOL animals (days 4–day 8: *p* = 0.003; days 4–14: *p* = 5.11 × 10^−4^) and SEN animals (days 4–14: *p* = 0.025). T-ES 7 showed a significant time effect (*p* = 0.007), with genetic line effect *p* = 0.812, interaction *p* = 0.811, and maximum Cohen’s *d* = 0.303. Temporal contrasts revealed significant changes in SEN animals (days 4–8: *p* = 0.040; days 4–14: *p* = 0.007). Other ES showed varying patterns without achieving statistical significance. T-ES 2 (genetic lines *p* = 0.812, time *p* = 0.430, interaction *p* = 0.641, maximum Cohen’s *d* = 0.405) and T-ES 3 (genetic line *p* = 0.812, time *p* = 0.065, interaction *p* = 0.977, maximum Cohen’s *d* = 0.137) exhibited small effect sizes. T-ES 4 (genetic line *p* = 0.812, time *p* = 0.135, interaction *p* = 0.641, maximum Cohen’s *d* = 0.326) showed temporal contrasts in SEN animals approaching significance (days 4–8: *p* = 0.051; days 4–14: *p* = 0.051). T-ES 5 (genetic line *p* = 0.757, time *p* = 0.924, interaction *p* = 0.641, maximum Cohen’s *d* = 0.572) and T-ES 6 (genetic line *p* = 0.812, time *p* = 0.315, interaction *p* = 0.864, maximum Cohen’s *d* = 0.269) showed moderate to small effect sizes without significant differences. T-ES 8 exhibited the largest effect size among all ES examined (maximum Cohen’s *d* = 0.960), though the omnibus tests did not achieve significance (genetic line *p* = 0.757, time *p* = 0.582, interaction *p* = 0.237). The Cohen’s d at day 14 (*d* = -0.960, 95% CI: −1.80 to −0.10) suggests potentially meaningful differentiation between genetic lines at this timepoint; however, the wide confidence interval, which nearly spans zero, reflects the uncertainty inherent in the sample size, and this pattern should be interpreted as hypothesis-generating rather than confirmatory. Temporal contrasts showed a significant decrease in SEN animals from day 4 to day 14 (*p* = 0.044).

#### 3.4.4 Statistical analysis with phenotypes

**FIGURE 4:**
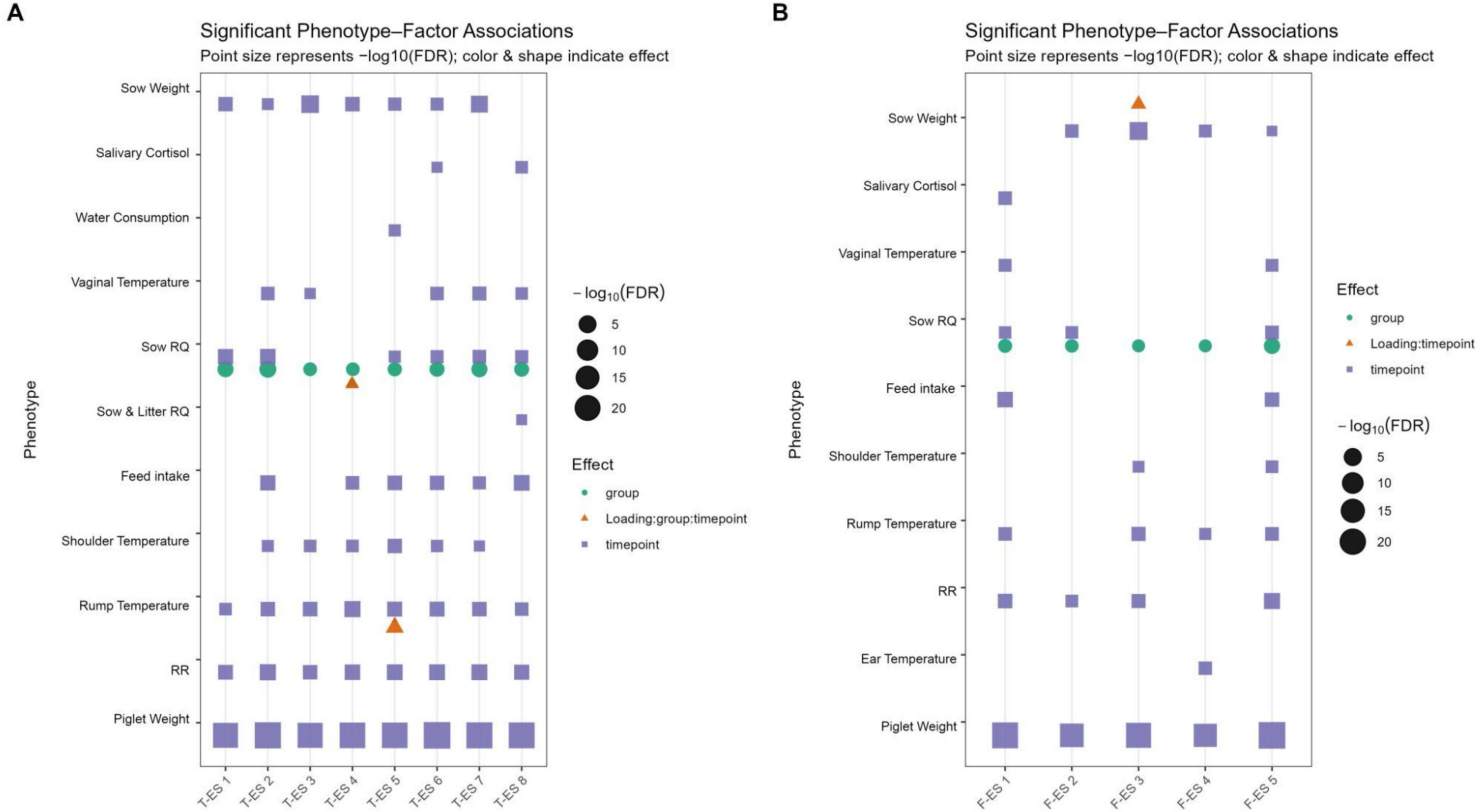
Significant phenotype–factor associations from MAG- and KO-based factor analyses. **(A)** Dot plot of MAG-based factor loadings. **(B)** Dot plot of KO-based factor loadings. In both panels, point size represents −log10(FDR), and point color and shape indicate the model effect (main, two-way, or three-way interactions). Points for the same phenotype–factor combination are vertically offset to avoid overlap.

Overall, ES–phenotype associations were largely driven by the independent contributions of time, genetic line, and ES load in the model. Temporal dynamics significantly influenced the phenotypes (12 out of 16), whereas genetic line effects were more restricted (4 out of 16). Two significant three-way interactions were observed: T-ES 5 interacting with RR, and T-ES 4 interacting with Sow–Litter RQ. Although TOL animals had higher predicted RR than SEN at day 4 (92.6 ± 4.05 vs. 85.9 ± 4.22), the group difference was not statistically significant. Both groups decreased over time, with TOL showing a stronger decline from day 4 to day 14 (-20.77 vs. −10.37 in SEN; *p* < 0.05), consistent with the significant three-way interaction. The latter showed a similar pattern: SEN exhibited higher predicted RQ than TOL on day 4 (1.33 ± 0.10 vs. 0.76 ± 0.07), with genetic line differences attenuating by day 14 (0.70 ± 0.06 vs. 0.80 ± 0.06; F*2*, 32.48 = 9.09, p_adj = 0.044). genetic line × phenotype interactions highlighted stronger ES–trait associations in TOL animals (e.g., T-ES 1 and T-ES 5 for Sow RQ: *F1*, _19_ = 46.08, p_adj = 7.48 × 10^−5^; *F1*_, 19_ = 23.11, p_adj = 2.61 × 10–3). Time × phenotype effects reflected longitudinal shifts in trait magnitude (e.g., RR associated with T-ES 5 decreased in TOL from 92.6 ± 4.05 at days 4–71.8 ± 4.11 at day 14; SEN: 85.9 ± 4.22 to 75.6 ± 4.66). T-ES 4 effects on Sow and Litter RQ also exhibited time-dependent changes (TOL: day 4 = 0.757 ± 0.075, day 14 = 0.797 ± 0.063) (Figure 4A and Table 1).

**TABLE 1:** Significant effects of ES on lactation phenotypes.

Phenotype	Factor	Effect	*F*_value	P_adj	Significance
Piglet weight	T-ES 6	Timepoint	408.4182	2.62 × 10^–25^	***
Piglet weight	T-ES 2	Timepoint	335.7509	4.56 × 10^–24^	***
Piglet weight	T-ES 7	Timepoint	333.248	1.13 × 10^–23^	***
Piglet weight	T-ES 8	Timepoint	378.7247	2.85 × 10^–23^	***
Piglet weight	T-ES 4	Timepoint	259.9097	1.63 × 10^–22^	***
Piglet weight	T-ES 5	Timepoint	261.8707	1.89 × 10^–22^	***
Piglet weight	T-ES 3	Timepoint	214.001	4.24 × 10^–21^	***
Piglet weight	T-ES 1	Timepoint	213.5741	7.05 × 10^–21^	***
Sow weight	T-ES 3	Timepoint	24.512	1.69 × 10^–6^	***
Sow RQ^1^	T-ES 2	Genetic line	75.01986	7.70 × 10^–6^	***
Sow weight	T-ES 7	Timepoint	20.27922	1.13 × 10^–5^	***
Sow RQ^1^	T-ES 7	Genetic line	51.56563	6.58 × 10^–5^	***
RR^2^	T-ES 2	Timepoint	16.14407	7.38 × 10^–5^	***
Sow RQ^1^	T-ES 1	Genetic line	46.07928	7.48 × 10^–5^	***
Temp temperature	T-ES 4	Timepoint	16.04222	7.75 × 10^–5^	***
RR^2^	T-ES 6	Timepoint	15.09648	1.37 × 10^–4^	***
RR^2^	T-ES 7	Timepoint	14.82654	1.37 × 10^–4^	***
Feed intake	T-ES 8	Timepoint	14.82921	1.74 × 10^–4^	***
RR^2^	T-ES 5	Timepoint	14.56822	1.76 × 10^–4^	***
RR^2^	T-ES 4	Timepoint	13.27136	2.94 × 10^–4^	***
Sow RQ^1^	T-ES 2	Timepoint	34.53371	3.19 × 10^–4^	***
Sow RQ^1^	T-ES 1	Timepoint	16.29386	5.10 × 10^–4^	***
Feed intake	T-ES 2	Timepoint	12.08487	5.49 × 10^–4^	***
RR^2^	T-ES 8	Timepoint	11.58888	6.31 × 10^–4^	***
Sow RQ^1^	T-ES 6	Genetic line	31.65982	6.40 × 10^–4^	***
Sow RQ^1^	T-ES 8	Genetic line	29.70158	7.53 × 10^–4^	***
Temp temperature	T-ES 6	Timepoint	11.06621	8.32 × 10^–4^	***
RR^2^	T-ES 1	Timepoint	10.41331	0.001	**
Feed intake	T-ES 5	Timepoint	10.0838	0.002	**
Temp temperature	T-ES 3	Timepoint	9.717872	0.002	**
Sow weight	T-ES 4	Timepoint	9.731145	0.002	**
Temp temperature	T-ES 5	Timepoint	9.696046	0.002	**
Temp temperature	T-ES 7	Timepoint	9.662474	0.002	**
Temp temperature	T-ES 2	Timepoint	9.399226	0.002	**
RR^2^	T-ES 3	Timepoint	9.224754	0.002	**
Feed intake	T-ES 6	Timepoint	9.332873	0.002	**
Shoulder temperature	T-ES 5	Timepoint	9.135809	0.002	**
Sow weight	T-ES 1	Timepoint	9.020653	0.002	**
Sow RQ^1^	T-ES 5	Genetic line	23.11287	0.003	**
Vaginal temperature	T-ES 7	Timepoint	8.814151	0.004	**
Sow RQ^1^	T-ES 3	Genetic line	19.66687	0.005	**
RR^2^	T-ES 5	Loading:genetic line:timepoint	13.0098	0.006	**
Vaginal temperature	T-ES 2	Timepoint	7.760677	0.007	**
Vaginal temperature	T-ES 6	Timepoint	7.639041	0.007	**
Temp temperature	T-ES 8	Timepoint	7.22453	0.007	**
Feed intake	T-ES 4	Timepoint	7.260104	0.007	**
Sow RQ^1^	T-ES 7	Timepoint	20.61315	0.007	**
Sow weight	T-ES 5	Timepoint	6.743547	0.009	**
Feed intake	T-ES 7	Timepoint	6.48746	0.011	*
Sow RQ^1^	T-ES 8	Timepoint	7.559901	0.012	*
Sow RQ^1^	T-ES 6	Timepoint	7.413837	0.012	*
Sow weight	T-ES 6	Timepoint	6.219512	0.013	*
Sow RQ^1^	T-ES 4	Genetic line	15.21038	0.015	*
Shoulder temperature	T-ES 4	Timepoint	5.749827	0.018	*
Salivary cortisol	T-ES 8	Timepoint	5.686168	0.019	*
Temp temperature	T-ES 1	Timepoint	5.636903	0.019	*
Shoulder temperature	T-ES 3	Timepoint	5.536991	0.019	*
Vaginal temperature	T-ES 8	Timepoint	5.771996	0.020	*
Shoulder temperature	T-ES 6	Timepoint	5.280172	0.023	*
Water consumption	T-ES 5	Timepoint	5.253863	0.026	*
Shoulder temperature	T-ES 2	Timepoint	5.049825	0.027	*
Sow RQ^1^	T-ES 5	Timepoint	5.716065	0.028	*
Sow weight	T-ES 2	Timepoint	4.832691	0.031	*
Vaginal temperature	T-ES 3	Timepoint	4.675614	0.039	*
Salivary cortisol	T-ES 6	Timepoint	4.338776	0.040	*
Shoulder temperature	T-ES 7	Timepoint	4.458497	0.041	*
Sow and litter RQ^1^	T-ES 8	Timepoint	4.485509	0.043	*
Sow and litter RQ^1^	T-ES 4	Loading:genetic line:timepoint	9.087082	0.044	*

^1^RQ, respiratory quotient.

^2^RR, respiratory rate. For each phenotype, the associated T-ES, factor tested (timepoint), F statistic, and adjusted *p*-value (P_adj) are reported. Only effects surviving multiple-testing correction are shown.

Significance levels are indicated as ***, **, or *.

### 3.5 KO analysis results

#### 3.5.1 F-ES composition

Five F-ES derived from KEGG Orthologs were mapped to their corresponding MAGs, displaying distinct KO compositions encompassing a wide range of metabolic and cellular functions. Similarly to T-ES compositions, ES loading denotes the contribution of a MAG to an ES, enrichment its relative overrepresentation, and specificity its exclusivity to that ES. F-ES 1 comprised 30 KOs across 13 functional categories, dominated by transport processes (5 KOs; total loading = 49.9%), unclassified functions (5 KOs; total loading = 51.0%), and transferases (4 KOs; total loading = 40.0%). Top contributors included K01872 (alanyl-tRNA synthetase; loading = 0.236), K02600 (two-component system sensor kinase; loading = 0.234), and K03701 (ATP-dependent DNA helicase; loading = 0.234), reflecting activities in protein synthesis, signal transduction, and DNA maintenance. F-ES 2 contained 30 KOs across 10 functional categories, with strongest representation in unclassified functions (12 KOs; mean plot value = 5.0), oxidation-reduction processes (5 KOs), and transferases (4 KOs). The top KOs were K00525 (ferredoxin-nitrite reductase; loading = 0.173), K02886 (ribosomal protein L2; loading = 0.172), and K02982 (ribosomal protein S3; loading = 0.172), indicating highly specific, uniform factor membership. F-ES 3 represented the largest and most functionally diverse ES, encompassing 658 KOs across 26 categories. Main contributions included unclassified functions (188 KOs; total loading = 2.53), regulation (115 KOs; 1.54), and transport (69 KOs; 0.93), with additional representation in oxidation-reduction (63 KOs; 0.85), kinases (24 KOs; 0.32), and transferases (24 KOs; 0.32). Top KOs were K03574 (7,8-dihydro-8-oxoguanine triphosphatase; 0.048), K06287 (uncharacterized protein; 0.047), and K00262 (glutamate dehydrogenase; 0.045), highlighting the broad functional scope of this ES. F-ES 4 comprised 30 KOs spanning 13 functional categories, dominated by unclassified functions (7 KOs; mean plot value = 4.94) and protein secretion systems (5 KOs; mean plot value = 4.96), with additional representation from regulation (4 KOs), transferases (3 KOs), and degradation processes (2 KOs). Top KOs included several uncharacterized proteins and secretion system components. F-ES 5 contained 44 KOs across 13 categories, dominated by oxidation-reduction processes (12 KOs; total loading = 0.297; mean plot value = 4.97) and unclassified functions (12 KOs; loading = 0.204). Other notable categories included transferases (5 KOs; 0.144), lipid metabolism (3 KOs; loading = 0.058), and regulation (3 KOs; loading = 0.033). Key KOs included K06118 (UDP-sulfoquinovose synthase; loading = 0.056) and K02259 (heme a synthase; loading = 0.053), suggesting specialized metabolic activities (Supplementary Table 3).

#### 3.5.2 Temporal and genetic line patterns of F-ES

F-ES distribution from the KO-based analysis varied between genetic lines and across the HS timeline (Figure 1B). Across the entire dataset, F-ES 1 was detected in 22 samples (8 SEN, 14 TOL), F-ES 2 in 15 samples (9 SEN, 6 TOL), F-ES 3 in 3 samples (3 SEN, 0 TOL), F-ES 4 in 31 samples (14 SEN, 17 TOL), and F-ES 5 in 3 samples (1 SEN, 2 TOL). F-ES 4 thus represented the most prevalent functional profile, present in 42% of samples. On day 4 (early HS phase), SEN animals exhibited relatively balanced factor representation, with F-ES 1 accounting for 33.3% of samples, F-ES 2 for 25.0%, F-ES 3 for 8.3%, and F-ES 4 for 33.3%. TOL animals demonstrated a distinct initial distribution, with F-ES 1 dominating at 53.8% of samples, while F-ES 2, 4, and 5 each represented 15.4% of samples. F-ES 3 was notably absent in TOL animals at this timepoint. By day 8, compositional patterns shifted in both genetic lines: in SEN animals, F-ES 4 became dominant at 41.7% of samples, followed by F-ES 2 (33.3%), F-ES 1 (16.7%), and F-ES 5 (8.3%), with F-ES 3 absent at this timepoint. On the other hand, in TOL animals F-ES 4 increased substantially to 53.8% of samples, while F-ES 1 decreased to 30.8%, and F-ES 2 remained stable at 15.4%. F-ES 3 and 5 were absent in TOL animals on day 8. On day 14, the convergence toward F-ES 4 became more pronounced in both genetic lines. In SEN animals, F-ES 4 represented 45.5% of samples, with F-ES 1 and 2 each accounting for 18.2% of samples and F-ES 3 reappearing at 18.2%. In TOL animals, F-ES 4 reached its highest representation at 61.5% of samples, while F-ES 1 decreased further to 23.1%, F-ES 2 remained at 15.4%, and F-ES 3 and 5 remained absent. This temporal progression indicates a systematic shift toward the F-ES 4 functional profile in both lines, with TOL animals showing more pronounced and consistent convergence. Interestingly, unlike the divergent taxonomic trajectories revealed in the MAG-derived T-ES analyses, with T-ES 1 becoming dominant in TOL animals while multiple ES persisted in SEN animals, this subsequent KO-based functional analyses showed a clear convergence of functional profiles.

#### 3.5.3 Statistical analyses

Across the five F-ES, linear mixed-effects models revealed minimal evidence of genetic line- or interaction-driven differences, with temporal dynamics generally absent (Figures 3C,D). In detail, F-ES 1 showed no significant effects (genetic line *p* = 0.582, time *p* = 0.544, genetic line × time *p* = 0.843), with a small effect size. F-ES 2 similarly lacked significance (genetic line *p* = 0.280, time *p* = 0.961, genetic line × time *p* = 0.788), and effect sizes were negligible. F-ES 3 exhibited a marginal time effect (time p_adj = 0.079), with genetic line *p* = 0.838 and genetic line × time *p* = 0.946; temporal contrasts suggested a weak trend toward longitudinal modulation but did not reach significance. F-ES 4 (genetic line *p* = 0.922, time *p* = 0.127, genetic line × time *p* = 0.882) and F-ES 5 (genetic line *p* = 0.582, time *p* = 0.807, genetic line × time *p* = 0.788) showed no significant effects, with small effect sizes across all factors. Overall, F-ES displayed minimal temporal or genetic line-specific variation under the HS conditions analyzed.

#### 3.5.4 Statistical analyses with phenotypes

Overall, F-ES–phenotype associations were dominated by single-factor effects, with temporal dynamics driving most responses. Time significantly influenced 13 of the 16 phenotypes across F-ES, whereas genetic line effects were comparatively limited, emerging for only a subset of traits (notably Sow RQ for F-ES 1–5). In contrast to T-ES, no significant three-way interactions (Loading × genetic line × timepoint) were detected, indicating that functional ES did not differentially shape phenotypic trajectories between TOL and SEN animals. Temporal modulation was widespread: all phenotypes except tail temperature, THP Sow-Only, and THP Sow–Litter exhibited significant time effects. For example, piglet weight increased consistently from day 4 to day 14 in both genetic lines under F-ES 1 (1.83 ± 0.08–3.85 ± 0.12 in TOL; 1.94 ± 0.08–3.86 ± 0.12 in SEN), a pattern replicated under F-ES 2–4 with overlapping confidence intervals. Similar longitudinal trends were evident for multiple temperature-derived traits, including vaginal, shoulder, rump, and ear temperatures, where both lines showed parallel declines or increases over time without genetic line-specific divergence. Genetic line × phenotype interactions were limited but, when present, reflected stable between-line differences rather than dynamic modulation, exemplified by the marked genetic line effect on Sow RQ for F-ES 5 (*F1*, 19 = 50.4, p_adj = 7.48 × 10^−5^). Only one Loading × timepoint interaction reached significance (Sow Weight for F-ES 3), indicating minimal time-dependent modulation of phenotypes by F-ES expression (Table 1 and Figure 4B).

### 3.6 Integrated patterns of T-ES and F-ES

#### 3.6.1 Cross-layer correlation and conceptual mapping

**FIGURE 5:**
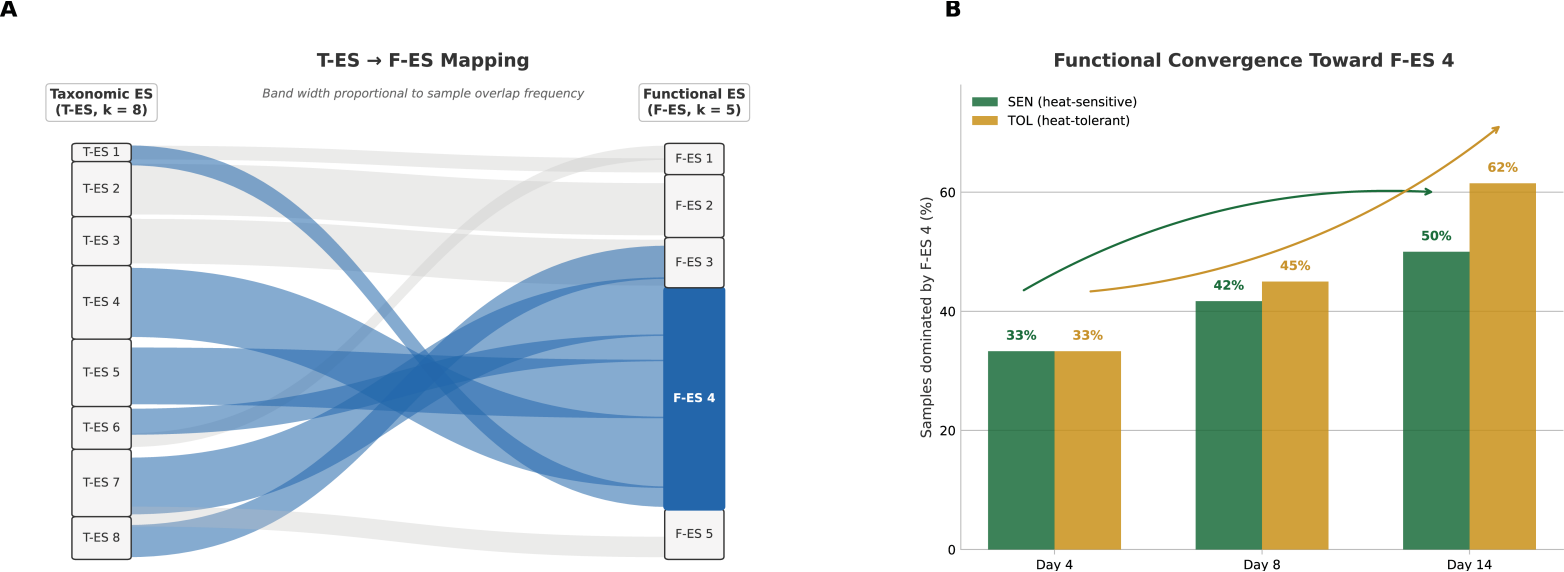
Taxonomic-to-functional ES mapping. **(A)** Sankey diagram illustrating the cross-layer correspondence between T-ES (k = 8, left) and F-ES (k = 5, right), based on pairwise Spearman rank correlations between NMF-derived sample score vectors across all 74 samples. Band width is proportional to co-occurrence frequency across samples. The dominant mapping pattern converges on F-ES 4, with contributions from multiple taxonomic states (T-ES 1, T-ES 4, and T-ES 7), providing visual evidence of functional redundancy. **(B)** Temporal dynamics of F-ES 4 dominance across lactation days 4, 8, and 14, stratified by genetic line. The proportion of samples dominated by F-ES 4 increased progressively in both heat-sensitive (SEN, green) and heat-tolerant (TOL, gold) sows, with TOL animals showing a steeper trajectory and reaching 62% dominance by day 14, compared to 50% in SEN animals.

To establish a conceptual framework for the integration of taxonomic and functional layers, we performed a Spearman correlation analysis between the sample loadings of the 8 T-ES and 5 F-ES (Figure 5). This cross-layer mapping revealed a spectrum of community organizational strategies, ranging from highly specific “taxa-function” pairings to broad functional redundancy. The most rigid correspondence was observed between T-ES 3 and F-ES 3 (ρ = 0.98, *p* < 0.001), indicating that the extreme taxonomic simplification (*Lactobacillus* dominance) and high functional breadth defining this state are intrinsically linked. In contrast, the dominant adaptive profile, F-ES 4, exhibited significant positive correlations with multiple taxonomic signatures, including T-ES 1 (ρ = 0.51), T-ES 4 (ρ = 0.72), and T-ES 7 (ρ = 0.30) (all *p* < 0.01). This many-to-many mapping provides statistical evidence for functional redundancy, demonstrating that the convergent functional state observed during heat stress can be sustained by diverse taxonomic assemblages. Conversely, F-ES 5 showed a high specific correlation with T-ES 2 (ρ = 0.66, *p* < 0.001), the signature characterized by opportunistic pathobionts. These correlations serve as the basis for the following detailed integration of the two analytical frameworks.

#### 3.6.2 Statistical sensitivity and effect size patterns

The two analytical approaches exhibited markedly different statistical patterns despite examining the same sample set. The MAG-based analysis detected significant temporal effects for two ES (T-ES 1: *p* = 5.42 × 10^−4^, Cohen’s *d* = 0.723; T-ES 7: *p* = 0.007, Cohen’s *d* = 0.303) and yielded a maximum effect size substantially larger than any observed in the functional analyses (T-ES 8: Cohen’s *d* = 0.960). In contrast, the KO-based analyses detected no significant effects of genetic lines, time, or their interaction across all 5 factors, with effect sizes ranging from Cohen’s *d* = 0.162 (F-ES 1) to *d* = 0.638 (F-ES 3). This divergence in statistical outcomes suggests that taxonomic composition exhibited greater jativity to HS effects than functional gene content. The detection of significant temporal changes in T-ES 1 and 7, coupled with larger maximum effect sizes, indicates that community taxonomic structure underwent measurable shifts during the HS period, whereas functional capacity remained relatively stable. This pattern is consistent with ecological principles of functional redundancy, wherein different taxonomic assemblages maintain similar functional capabilities, allowing functional stability despite taxonomic turnover. The relationship between effect size magnitude and statistical significance also differed between analyses. In the KO analyses, moderate effect sizes (e.g., F-ES 3: Cohen’s *d* = 0.638) did not achieve significance, whereas in the MAG analyses, smaller effect sizes (T-ES 7: Cohen’s *d* = 0.303) reached statistical significance. This discrepancy likely reflects differences in the variance structure and distributional characteristics between T-ES and F-ES scores, as well as the greater number of ES in the MAG analyses potentially reducing statistical power for any single ES while increasing overall sensitivity to temporal change.

#### 3.6.3 Temporal dynamics: convergence versus divergence

The T-ES and F-ES analyses revealed contrasting temporal trajectories that show different aspects of community response to HS. To formally test these patterns, we computed between-group centroid distances in NMF W-matrix space at each timepoint and assessed directional change via permutation testing (9,999 permutations; Supplementary Figure 2). In functional ES space, between-group centroid distances were near zero across all timepoints (D4 = 0.002, D8 = 0.001, D14 = 0.002; Δ = -4 × 10^–5^, p(convergence) = 0.684), indicating that TOL and SEN animals maintained functionally equivalent community states throughout the HS period rather than converging from initially distinct positions. PERMANOVA confirmed that genetic line explained negligible functional variance at any timepoint (*R*^2^ = 0.039–0.068, all *p* > 0.18). In contrast, taxonomic ES showed increasing between-group centroid distances from D4 (0.54) through D8 (0.61) to D14 (0.78; Δ = 0.247, p(divergence) = 0.097), a directional trend approaching significance. PERMANOVA *R*^2^ for genetic line in taxonomic space increased over time (0.025 → 0.032 → 0.076), approaching significance at D14 (*p* = 0.110). The taxonomic analysis revealed divergent trajectories between genetic lines: TOL animals exhibited a marked shift toward dominance of T-ES 1 (increasing from 0% at day 4 to 38.5% at day 14, *p* = 5.11 × 10^−4^), while SEN animals showed more heterogeneous patterns with elevated representation of T-ES 8 at day 14 (27.3% of samples, Cohen’s d = 0.960). The maintained functional equivalence, combined with the trend toward taxonomic divergence, suggests that the two genetic lines maintained similar functional states through distinct and increasingly divergent taxonomic pathways, consistent with functional redundancy operating at the community level. Different taxonomic assemblages in TOL and SEN animals achieved similar functional capacities, suggesting that the microbiome’s adaptive response to HS prioritized maintenance of core metabolic functions while permitting taxonomic flexibility. This pattern implies that functional stability, rather than taxonomic stability, may represent the key feature of resilient microbiome responses to environmental perturbation.

#### 3.6.4 Ecologically imbalanced ES and community states

Both analytical approaches identified ecologically imbalanced community states, though they emphasized different aspects of community imbalance. F-ES 3, detected in 3 samples exclusively from SEN animals at days 4 and 14, exhibited the highest functional diversity (658 KO across 26 categories) coupled with the largest effect size among F-ES (Cohen’s *d* = 0.638). Its occurrence only in SEN animals and absence on day 8 is consistent with a genotype-associated pattern under HS exposure. When looking at their taxonomy, these same samples corresponded to T-ES 3, which exhibited extreme taxonomic simplification with 88% of total loading contributed by a single taxon (*Lactobacillus amylovorus* MAG021, enrichment = 7.810). While such dominance reflects marked community simplification, *Lactobacillus* prevalence is not inherently pathological in all contexts and should therefore be interpreted cautiously. This combination of high functional variation with extreme taxonomic dominance represents the most pronounced low-diversity configuration detected in the dataset. The community state characterized by enrichment of taxa with opportunistic potential, identified as T-ES 2 (15 samples, 20% of dataset) showed highly specific functional membership (mean plot values approaching 5.0 across categories) with moderate effect size (Cohen’s *d* = 0.500). The corresponding taxonomic pattern in T-ES 2 revealed enrichment of multiple taxa frequently described as opportunistic or context-dependent pathobionts in vertebrate microbiomes, including *Corynebacterium* species (three MAGs with enrichments 7.991, 7.745, and 7.126), *Enterococcus_B faecium* (enrichment = 6.923), *Helicobacter_A rappini* (enrichment = 5.686), and *Escherichia coli* (enrichment = 3.937). The MAG analysis thus provided greater resolution of the specific pathobiont taxa contributing to this dysbiotic state, while the KO analysis captured the distinctive functional ES associated with their presence. T-ES 8, which showed the largest effect size in the entire study (Cohen’s *d* = 0.960), did not reach statistical significance and was associated with wide uncertainty. This ES, elevated in SEN animals at day 14, included diverse Actinomycetota (6 MAGs) and Campylobacterota (3 MAGs including *Helicobacter_F*, *Campylobacter_B*, and *Campylobacter_D* species), suggesting a distinct taxonomically enriched configuration observed in SEN animals under prolonged HS exposure. The absence of a corresponding discrete F-ES suggests that this taxonomic configuration’s functional attributes overlapped with other functional ES, possibly indicating that taxonomic analysis can detect biologically meaningful community states not distinguishable in functional space. Given the limited sample representation of some ES and the compositional nature of the data, interpretations of dysbiosis-associated configurations should be considered biologically suggestive rather than definitive.

#### 3.6.5 Balanced and health-associated ES

In contrast to the dysbiotic configurations, both analytical layers also identified community states consistent with balanced structure and host-associated gut health. F-ES 4, the most prevalent functional ES (31 samples, 42% of dataset), showed moderate functional specificity with emphasis on protein secretion systems and regulatory functions (Cohen’s *d* = 0.575). The temporal convergence toward this ES in both genetic lines, particularly pronounced in TOL animals (61.5% of samples at day 14), suggests it represents an adaptive functional state favorable for coping with HS. This functional ES corresponded most closely to T-ES 4 and 5, both dominated by Bacillota_A and characterized by balanced taxonomic compositions. T-ES 4 included health-associated taxa such as *Faecalibacterium prausnitzii_I* (enrichment = 7.916) and diverse Oscillospirales members, while T-ES 5 was enriched in fiber-degrading specialists including *Ruminococcus flavefaciens_E* and *R. flavefaciens_T* (enrichments 7.737 and 7.465, respectively) along with *Christensenella minuta* (enrichment = 7.999). The correspondence between a single dominant F-ES and multiple T-ES illustrates functional convergence through distinct taxonomic paths, with different combinations of Bacillota_A-dominated communities achieving similar functional capabilities. The significant increase in T-ES1 in TOL animals (days 4–14: *p* = 5.11 × 10^−4^) represents an additional balanced community state characterized by broad phylogenetic diversity (9 phyla) including Bacteroidota, Bacillota, Bacillota_A, Fibrobacterota, Spirochaetota, and Verrucomicrobiota representatives. This ES’s strong association with the TOL phenotype and its temporal increase during HS suggests it may represent an adaptive taxonomic configuration. The absence of a discrete corresponding F-ES indicates that this taxonomic pattern’s functional characteristics overlapped with the broader T-ES 4 profile, further supporting the principle that multiple taxonomic configurations can yield similar functional capabilities.

#### 3.6.6 Additional community variants

The MAG analysis identified 3 additional ES (T-ES 6, 7, and 8) representing taxonomic variants not resolved as discrete factors in the KO analysis. For example, T-ES 6 exhibited specificity for *Porphyromonas* and *Prevotella* genera, with top enriched MAGs achieving enrichment values of 8.000 and perfect specificity scores (1.000), while T-ES 7 showed dominance of Oscillospirales with multiple *Faecousia* species representatives. These ES likely represent subtle functional variations that were subsumed within broader KO-based factors, demonstrating that major taxonomic differences can correspond to relatively minor functional differences. The ability of taxonomic analysis to detect these variants highlights its value as a complementary approach to functional profiling, particularly for identifying community states that may have ecological or clinical relevance despite functional similarity.

#### 3.6.7 Sample-level correspondence and community organization

Examining sample-level assignments across the two frameworks showed partial but imperfect overlap between F-ES and T-ES, offering insight into how communities are organized. Samples classified as F-ES 3 almost always matched T-ES 3, indicating a tight link between extreme functional breadth and the marked taxonomic simplification that define this dysbiotic state. Likewise, most F-ES 2 samples corresponded to T-ES 2, although some with this functional profile appeared in other T-ES, indicating that pathobiont-associated functions can emerge from different taxonomic backgrounds. In contrast, F-ES 4 samples were spread across multiple T-ES, mainly T-ES 4, 5, and 7. This many-to-many mapping between functional and taxonomic ES suggests that the balanced functional profile of F-ES 4 can be supported by several distinct taxonomic configurations. Such functional equivalence among taxonomically different communities helps explain the observed pattern of functional convergence despite taxonomic divergence during HS. This partial decoupling of functional and taxonomic ES was also reflected in the lack of one-to-one correspondence in significant effects. T-ES 1 increased strongly over time in TOL animals (*p* = 5.11 × 10^−4^), but no individual F-ES showed a parallel temporal change in this line.

## 4 Discussion

The present study leveraged NMF to dissect the ecological structure of the gut microbiome in lactating sows under HS conditions, moving beyond static taxonomic descriptions to identify latent microbial subcommunities that capture coordinated community behavior and reveal how microbiomes reorganize in association with host physiological responses.

### 4.1 Interpretable microbial community structure through NMF

NMF enabled decomposition of microbiome data to provide robust latent factors for both T-ES and F-ES profiles, offering interpretable community signals that can be assigned to individual samples or combined into experimental units depending on the analytical needs (Jiang et al., 2004; Jiang et al., 2017; Breuninger et al., 2021). Factorization ranks differed between taxonomic (*k* = 8) and functional (*k* = 5) data, likely reflecting differences in analytical resolution: taxonomic composition captured more granular community variants, whereas functional redundancy grouped taxa into fewer ES because functional gene repertoires converge due to metabolic constraints and shared ecological requirements (Louca et al., 2016, 2018). Also, it should be considered that taxonomic composition is more variable due to stochastic colonization, priority effects, and phylogenetic trait distributions (Martiny et al., 2015). Nevertheless, the factorization results were in both cases robust (cophenetic correlations > 0.95, silhouette consensus > 0.95) and adequately validated. External subsampling validation confirmed that taxonomic ES were consistently recovered (mean Jaccard = 0.67; 6 of 8 signatures ≥ 0.60), while functional ES showed mixed but defensible stability, with four of five signatures reproducible and one (F-ES 3) demonstrating limited stability due to low prevalence. Sensitivity analyses at adjacent ranks (*k* ± 1) yielded qualitatively similar ES compositions, supporting robustness of biological interpretation. Using the NMF approach allowed the identification of gradual shifts in community structure under HS, an insight linked to continuous mixing proportions rather than discrete ET (Frioux et al., 2023). It must be acknowledged that NMF does not account for phylogenetic relationships and requires careful compositional correction (Armstrong et al., 2022). To date, no current dimensionality-reduction method simultaneously provides phylogenetic awareness, compositional handling, and clear interpretability, so complementary approaches remain necessary. In this study, NMF provided interpretable community ES for comparing TOL versus SEN animals while accepting trade-offs in phylogenetic resolution.

### 4.2 Divergent microbiome trajectories between TOL and SEN animals

Although both genetic lines converged toward similar functional profiles (F-ES 4; 61.5% in TOL vs. ∼50% in SEN at day 14), their taxonomic communities diverged sharply over time. TOL animals progressively shifted toward phylogenetically diverse and balanced communities (T-ES 1: 0–38.5% from days 4 to 14), whereas SEN animals transitioned into ecologically imbalanced states marked by enrichment of taxa with pathobiont potential (T-ES 8; Cohen’s d = 0.960) or single-taxon dominance (T-ES3: 88% *Lactobacillus amylovorus*). An illustrative example is T-ES 4, enriched for protein secretion systems and regulatory functions (Lara and Rostagno, 2013; Kim et al., 2022), of which its temporal realization differed between genetic lines, with TOL animals assembling phylogenetically diverse, functionally redundant consortia, whereas SEN animals relied on taxonomically restricted, compositionally unstable configurations. This dissociation between functional convergence and taxonomic divergence suggests that while F-ES 4 may represent a common functional endpoint under HS, its association with tolerance could depend on the taxonomic pathway by which it is reached. The most pronounced genetic line differences were evident in these temporal dynamics: in TOL animals, T-ES 1 spanning nine phyla including *Faecalibacterium prausnitzii* and *Ruminococcus flavefaciens* (Lopez-Siles et al., 2017; Lindstad et al., 2021; Martín et al., 2023), alongside maintained or rising phylogenetic diversity, indicating recruitment of additional taxa rather than species loss. SEN animals instead followed trajectories not observed in TOL animals that may reflect less stable or transitional community states: competitive exclusion by *L. amylovorus* (T-ES3), early enrichment of opportunists such as *Enterococcus faecium*, *Corynebacterium*, *Helicobacter rappini*, and *Escherichia coli* (T-ES 2) that exploit compromised barriers (Torres et al., 2018; Gião et al., 2022; Liu et al., 2023), and late-stage progression to a suboptimal equilibrium dominated by *Actinomycetota* and *Campylobacterota* (T-ES 8). Shared ES further highlighted these divergent trajectories: T-ES 4, 5, and 7, Bacillota_A-dominated, and fiber-degrading communities occurred in both genetic lines but followed distinct developmental patterns. In TOL animals they preceded or co-occurred with the transition into stable, multi-phylum T-ES 1, whereas in SEN animals they appeared transiently and failed to progress toward more resilient configurations. By contrast, the three ecologically imbalanced ES (T-ES 2, 3, and 8) were exclusive to SEN animals, possibly reflecting community configurations associated with reduced resilience rather than clinically defined dysbiosis. Imbalanced ES were identified by highly uneven taxonomic loadings, with dominance by a limited number of taxa rather than a broadly distributed microbial composition, suggesting that TOL animals may exhibit host or microbiome features that reduce the likelihood of transitioning into these states under HS. The emergence of T-ES 1 in TOL animals likely reflects a high-redundancy architecture where multiple taxa contribute to each functional category, providing a kind of “insurance” against species loss (Allison and Martiny, 2008; Cross et al., 2025). Recent demonstrations that functional redundancy averages approximately 0.4 across body sites (Kim et al., 2022) support this interpretation, with the nested network structure, low functional distances between taxa, and heterogeneous gene degree distribution characteristic of high-redundancy (Tian et al., 2020), align with the multi-phylum composition of T-ES 1. SEN animals’ patterns, characterized by single-taxon dominance or pathobiont enrichment, may reflect lower-redundancy architectures that could be more vulnerable to functional instability: they can reach the functional endpoint (F-ES 4 enrichment) but lack the structural stability to maintain it.

### 4.3 Linking microbiome dynamics to host phenotypes

A pattern like the divergence between functional convergence and taxonomic trajectories, where temporal dynamics shaped the progression of ES differently in TOL and SEN animals, emerged when linking ES to host phenotypes. Across both taxonomic and functional spaces, temporal dynamics accounted for most of the phenotypic variation, whereas genetic line effects and higher-order interactions were comparatively limited. This aligns with established observations that heat-stressed lactating sows exhibit strong time-dependent physiological adjustments over the course of lactation, regardless of genetic background (Johnson et al., 2019; Byrd et al., 2025). In our framework, functional ES exerted largely uniform effects across TOL and SEN animals, with no significant three-way interactions and only isolated genetic line × phenotype differences (e.g., Sow RQ under F-ES5). This pattern is consistent with the possibility that functional redundancy contributes to convergence at the metagenomic level and may be associated with broadly similar temporal phenotypic profiles, consistent with the idea that metabolic demands of lactation, rather than genetic line identity, govern many of the HS responses: however, in the absence of a non-heat-stressed control group, this interpretation remains hypothetical. In contrast, T-ES revealed a more nuanced interaction with host physiology: although single-ES effects remained predominant, a subset of T-ES exhibited genetic line-specific temporal modulation, particularly for traits associated with thermoregulation and metabolic heat production. These findings are alike to prior reports demonstrating that TOL sows maintain greater THP early in lactation and rely on distinct thermoregulatory strategies, including elevated latent heat dissipation via RR, limit body temperature escalation under HS conditions (DeShazer et al., 2009; Byrd et al., 2025). The ES-based associations refine this interpretation by showing that only specific taxonomic configurations (not functional ones) diverge between TOL and SEN animals in ways that affect early-lactation heat loss capacity and substrate oxidation patterns (e.g., interactions with RR and Sow–Litter RQ). Importantly, these dynamics were transient and diminished by the end of the trial on day 14, paralleling earlier physiological evidence that TOL advantages in THP are most pronounced when milk production demands are still relatively low. Taken together, these results are consistent with a model in which functional convergence corresponds to shared metabolic constraints across genetic lines, producing broadly similar longitudinal phenotypes, whereas taxonomic composition governs the early-stage flexibility of thermoregulatory and metabolic responses that distinguish tolerance from sensitivity. This interpretation is consistent with emerging multi-omics literature demonstrating that microbiome-mediated resilience under thermal stress arises from early, coordinated host–microbe adjustments rather than persistent differences in steady-state function (Gini et al., in prep). It further raises the possibility that selection for heat tolerance may interact with microbiome structure, particularly through the taxonomic routes by which sows reach common functional states, a pattern that may underpin the modest but cumulative gains expected in early generations of genomic selection for thermotolerance. Continued genomic selection may therefore amplify these taxonomically mediated advantages, enabling TOL animals to more effectively leverage conserved microbial functions during periods of intense metabolic demand.

### 4.4 Functional redundancy

The maintained equivalence of functional profiles (between-group distance ≈ 0 across all timepoints; permutation *p* = 0.684) despite a trend toward increasing taxonomic divergence (Δ = 0.247, *p* = 0.097) provides quantitative support for functional redundancy at the community scale. Similar patterns have been reported in human gut microbiomes, where functional gene repertoires remain relatively conserved despite extensive taxonomic variability (Qin et al., 2010; The Human Microbiome Project Consortium, 2012). Quantitative analyses estimate functional redundancy at approximately 0.4 across body sites, a balance attributed to nested community organization, low functional distances among taxa, and heterogeneous gene-degree distributions (Tian et al., 2020). In this context, the progressive enrichment of F-ES 4 in both genetic lines, particularly in TOL animals, suggests that this functional configuration may be selectively enriched under HS conditions. Its enrichment in protein secretion systems and regulatory functions may reflect coordinated community-level responses that support cellular maintenance and inter-microbial interactions during stress (Lara and Rostagno, 2013; Kim et al., 2022), potentially facilitating metabolite exchange under reduced nutrient availability (Yu et al., 2024). Our cross-layer correlation analysis directly supports this redundancy model; while F-ES 4 represented a common functional endpoint, it exhibited significant positive correlations with multiple, phylogenetically distinct taxonomic signatures, including T-ES 1, 4, and 7 (all *p* < 0.05). This “many-to-many” mapping demonstrates that the sow microbiome maintains functional stability through taxonomic flexibility, allowing diverse microbial assemblages to fulfill the same core metabolic roles. Theoretical frameworks predict that such redundancy enhances ecosystem stability by buffering essential functions against species loss (Allison and Martiny, 2008; Cross et al., 2025). The shift of TOL animals toward the phylogenetically diverse T-ES 1 is consistent with a higher-redundancy architecture in which multiple taxa contribute to shared functions. In contrast, the rigid correspondence between T-ES 3 and F-ES 3 (ρ = 0.98, *p* < 0.001) observed in SEN animals characterizes a low-redundancy configuration marked by taxonomic simplification. Such configurations may limit functional stability and increase vulnerability to environmental perturbation, even when a similar functional endpoint is temporarily achieved.

### 4.5 HS Mechanisms and dysbiosis mechanisms

HS imposes multiple constraints on the gut, including reduced splanchnic blood flow, impaired nutrient delivery to enterocytes, and activation of stress and inflammatory pathways (Chen et al., 2018; Zhu et al., 2019; He et al., 2020; Wang et al., 2022; Koch et al., 2024). These perturbations can weaken intestinal barrier integrity through structural damage to the epithelium, tight junction disruption, and increased paracellular permeability (Xia et al., 2022). Such changes are thought to reshape microbial community composition, typically through reorganization rather than complete species loss (Zhu et al., 2019; He et al., 2020; Ncho, 2025).

Across livestock species, HS-induced microbiome shifts share common features. A systematic review of 18 poultry studies found that alpha diversity remains largely stable while beta-diversity shifts, indicating structural reorganization (Ncho, 2025). Facultative anaerobes, especially Proteobacteria, often expand, whereas taxa such as *Faecalibacterium*, *Ruminococcus*, *Lactobacillus*, and *Bifidobacterium* decline (Seo et al., 2024; Ncho, 2025). In dairy cattle, baseline enterotype composition shapes these responses: heat tolerant cows retain functionally stable microbiomes, while more heat-sensitive cows show higher diversity but reduced functional stability (Li et al., 2025). In pigs, compositional shifts coincide with epithelial damage, and host genetics influences enterotype assembly (Xia et al., 2022; Mancin et al., 2024).

Within this context, the SEN-specific ES identified here may represent candidate manifestations of HS-associated dysbiosis. T-ES 3, dominated by *Lactobacillus amylovorus*, was highest at Day 4 and declined thereafter, is consistent with competitive exclusion and loss of commensal diversity; prior work suggests that *L. amylovorus* contributes to stable communities only at moderate abundance (Sarpong et al., 2024). T-ES 2, enriched in opportunistic taxa including *Enterococcus faecium* and *Escherichia coli*, peaked around Day 8, aligning with patterns reported following barrier compromise (Cheleuitte-Nieves et al., 2018; Torres et al., 2018; Gião et al., 2022; Liu et al., 2023). T-ES 8 emerged later in the HS cycle and reached its maximum at day 14, potentially reflecting a stable but suboptimal community state.

Although temporal effects dominated physiological variation in both genetic lines, early thermoregulatory differences previously reported in this population, particularly higher heat dissipation in TOL sows during early lactation, may have attenuated gut perturbation in these animals. The exclusive appearance of imbalanced ES in SEN animals is consistent with the possibility that TOL hosts possess buffering capacity,, potentially involving host genetics, immune function, or baseline microbiome composition, that limits transitions into dysbiotic states under equivalent environmental challenge.

### 4.6 Beneficial taxa enrichment in TOL animals

The temporal dynamics of T-ES 1 provide one of the clearest microbiome signatures distinguishing TOL from SEN animals. Its progressive increase from days 4 to 14 in TOL sows, contrasted with its absence in SEN animals, suggests progressive assembly of a stress-associated community configuration rather than passive preservation of baseline composition. Spanning nine phyla, T-ES1 represents a phylogenetically diverse and ecologically mature configuration that TOL animals progressively establish under HS, whereas SEN animals fail to transition into this state. Several taxa enriched within T-ES 1 offer mechanistic insight into its association with tolerance. *Faecalibacterium prausnitzii*, a cornerstone commensal of healthy mammalian guts (Lopez-Siles et al., 2017; Martín et al., 2023), contributes to butyrate production, epithelial energy supply, barrier maintenance, and immune modulation (Carlsson et al., 2013). Recent evidence indicates *that F. prausnitzii* exerts system-level effects through bioactive outer membrane vesicles, including metabolites such as phosphatidylcholine that reduce epithelial necroptosis and support barrier integrity (Xing et al., 2025). Fiber-degrading specialists, including *Ruminococcus flavefaciens* and *Christensenella minuta*, together with butyrate-producing Oscillospirales, likely sustain energy harvest and redox balance during HS when feed intake is reduced (Carlsson et al., 2013; Fagundes et al., 2021; Lindstad et al., 2021). Comparable patterns have been reported in more heat tolerant cattle, where dominance of *R. flavefaciens* coincides with enrichment of pentose phosphate pathway activity and enhanced oxidative stress management (Li et al., 2025). The correspondence between this taxonomically diverse configuration and the shared functional endpoint represented by F-ES 4, which is also achieved through alternative T-ES configurations (T-ES 4, 5, and 7), highlights a central theme of this study: resilience under HS is supported not by a single optimal composition but by taxonomic diversity and functional redundancy. T-ES 1 appears to represent a higher-redundancy architecture that may stabilize functional outputs while preserving flexibility in community assembly, a property absent from the trajectories observed in SEN animals.

### 4.7 Functional versus taxonomic profiling

Taxonomic and functional profiling yielded distinct statistical patterns, with T-ES showing strong temporal structure, followed by genetic line-specific trajectories, whereas functional ES exhibited comparatively limited differentiation. This discrepancy likely reflects fundamental biological differences between the two data layers. Taxonomic markers tend to exhibit higher heritability and clearer lineage-specific signals, whereas functional genes are frequently shared across taxa through horizontal gene transfer and are embedded in redundant metabolic networks (Lozupone et al., 2012; Boon et al., 2014). As a result, changes in community composition may be detected earlier and more sensitively at the taxonomic level than at the level of inferred functional potential. Nonetheless, functional profiling provided complementary insight by revealing convergence toward shared metabolic strategies despite divergent taxonomic trajectories. This pattern is consistent with prior work showing that functional profiles can remain relatively stable across individuals or conditions even when taxonomic composition varies substantially (Börnigen et al., 2013; Manor and Borenstein, 2017). At the same time, functional inference from metagenomes remains constrained by incomplete reference databases, and stress-responsive genes may remain unannotated or poorly characterized, limiting the detection of subtle but biologically meaningful functional shifts (Langille, 2018). Taken together, these findings underscore the value of integrating taxonomic and functional perspectives. Taxonomic profiling may serve as a sensitive indicator of ecological perturbation and early dysbiosis, whereas functional profiling helps contextualize how communities maintain or reorganize metabolic capacity in response to HS (Graham et al., 2016; Li et al., 2024) The observation that dysbiotic states could arise through taxonomic simplification or pathobiont enrichment with overlapping functional ES highlights the limitations of either approach in isolation. Future integration with additional omics layers, including metatranscriptomics, metabolomics, and host phenotyping, will be essential to resolve how genetic potential translates into realized function and to identify the mechanisms that ultimately underpin resilience under thermal stress (Muller et al., 2024; Duan et al., 2025; Yang S. Y. et al., 2025).

### 4.8 Limitations and alternative interpretations

Several constraints should be considered when interpreting microbiome–host associations in this study. The modest sample size (74 samples from 26 animals) limited temporal resolution (three timepoints over 14 days), and exclusive reliance on fecal sampling reduced statistical power, restricts generalizability, and may fail to capture microbial dynamics in proximal gut regions that more directly influence thermoregulation, nutrient absorption, and barrier function (Xia et al., 2022; Yang S. Y. et al., 2025). While the absence of a thermoneutral control group precludes definitive attribution of temporal shifts to HS alone, the divergent trajectories between TOL and SEN animals under identical environmental conditions support genetic line-specific responses to thermal challenge; however, future studies incorporating concurrent thermoneutral controls are warranted. Additionally, the study design cannot fully disentangle genetic line effects from potential confounding factors. TOL and SEN classifications were based on genomic estimated breeding values from a parent population, but physiological differences between lines (e.g., baseline metabolic rate, feed intake patterns, or pre-existing microbiome composition) could independently influence both HS responses and microbiome dynamics. The absence of pre-HS baseline samples further limits our ability to distinguish treatment effects from pre-existing genetic line differences. Moreover, the observational design precludes definitive causal inference: while differential ES trajectories aligned with host phenotypes such as the persistence of imbalanced ES in SEN animals with higher physiological strain (elevated temperature, respiration rate, and reduced performance), alternative explanations are possible. Host genetic or physiological factors could influence microbiome composition independently of microbiome-mediated effects on HS tolerance. Experimental manipulation, including fecal microbiota transplantation or gnotobiotic models, would be required to establish causality more robustly (Xia et al., 2022). Functional predictions derived from genomic content assume that gene presence reflects activity; however, post-transcriptional regulation, environmental conditions, and substrate availability may decouple potential from realized function (Zengler and Palsson, 2012; Li et al., 2023). Similarly, functional redundancy estimates are sensitive to methodological assumptions, and reductions in apparent functional variability may reflect statistical averaging rather than true selection on metabolic capacity. Integration with metatranscriptomics, metabolomics, or proteomics could provide more direct insight into active pathways and community function under HS. Together, these limitations highlight the need for cautious interpretation: while the observed ES patterns suggest mechanisms of resilience and vulnerability, additional experimental and multi-omics studies are necessary to validate their causal role in host adaptation to HS.

### 4.9 Future research directions

Further investigation is needed to understand the temporal and mechanistic dynamics of ES under HS. Longitudinal studies spanning multiple heat cycles would clarify whether communities’ cycle repeatedly through the same states or progressively drift toward distinct equilibria, providing insight into cumulative impacts of climate change on livestock microbiomes. Expanded sampling beyond fecal microbiota, especially small intestine and mucosa-associated communities, would provide a more complete picture of gut region-specific responses (Yang J. et al., 2025). Integration with multi-omics approaches, including metatranscriptomics, metabolomics, and host physiology measurements, will be critical for linking ES trajectories to functional mechanisms underpinning thermotolerance (Muller et al., 2024; Duan et al., 2025; Yang S. Y. et al., 2025). Importantly, the imbalanced ES exclusive to SEN animals should be evaluated for genetic underpinnings and heritability, as potential early indicators of heat susceptibility. Experimental validation is also necessary. Manipulative studies such as fecal microbiota transplantation or gnotobiotic models could establish causality between ES configurations and host resilience, clarifying whether observed trajectories actively contribute to tolerance or merely reflect host state. Mechanistic understanding of taxa-specific contributions: for instance, butyrate production by *F. prausnitzii* or pathobiont translocation under barrier compromise could guide precision nutrition and microbial interventions.

### 4.10 Major takeaways

This work introduces several contributions to understanding microbiome dynamics under HS in lactating sows. First, ES decomposition of longitudinal metagenomic data showed that data-driven subcommunity profiling can capture temporal microbiome reorganization patterns not evident from standard taxonomic summaries. Second, our findings indicate a decoupling between functional and taxonomic trajectories. While both genetic lines converged toward similar functional profiles (F-ES 4 enrichment), they did so through distinct taxonomic routes: TOL animals assembled diverse, balanced communities (T-ES 1), whereas SEN animals transitioned through ecologically imbalanced states (T-ES 2, 3, 8) marked by enrichment of taxa with pathobiont potential or competitive exclusion. This “many-to-many” mapping between taxa and function supports the hypothesis that functional redundancy provides a buffering mechanism for community-level resilience, where the specific taxonomic path to functional convergence differentiates adaptive from maladaptive outcomes. Third, the exclusive occurrence of imbalanced ES in SEN animals points to the potential utility of ES profiling for early identification of heat-susceptible individuals. If replicated and validated in larger, independent cohorts, such signatures could inform targeted interventions before productivity losses occur. These findings should be interpreted as hypothesis-generating given the study’s sample size and observational design, but they provide a framework for integrating microbiome structure with host phenotypes in precision livestock management under thermal challenge.

## Data Availability

The data presented in this study are publicly available. The data can be found at: https://www.ncbi.nlm.nih.gov/bioproject?term=PRJNA1320939, accession PRJNA1320939.
