# Supplementary material for: Data-driven enterosignatures link gut microbiome reorganization to heat stress responses in lactating sows
Source: Front Microbiol. 2026 Apr 10;17:1797687. doi: 10.3389/fmicb.2026.1797687 (PMC13108611; doi:10.3389/fmicb.2026.1797687)
Supplement: Supplementary file 1 [file Data_Sheet_1.docx]

Supplementary Material

# Supplementary Data

The datasets analyzed for this study can be found in the NCBI Sequence Read Archive under BioProject PRJNA1320939 (<https://www.ncbi.nlm.nih.gov/bioproject?term=PRJNA1320939>) .

# Supplementary Figures and Tables

## Supplementary Tables

**Supplementary Table 1:** Per-sample sequencing depth, assembly statistics, and MAG quality metrics.

| **Sample** | **Raw Reads (M)** | **QC Reads (M)** | **QC Retention (%)** | **MAGs Recovered** | **Mean N50 (kb)** | **Mean Completeness (%)** | **Mean Contamination (%)** | **HQ MAGs** |
| --- | --- | --- | --- | --- | --- | --- | --- | --- |
| S037 | 56.75 | 21.18 | 37.3 | 4 | 29.2 | 90.8 | 0.67 | 3 |
| S038 | 77.63 | 12.08 | 15.6 | 2 | 51.4 | 99.8 | 0.04 | 2 |
| S039 | 83.53 | 25.78 | 30.9 | 15 | 157.3 | 93.3 | 0.5 | 12 |
| S040 | 79.98 | 8.86 | 11.1 | 1 | 11.9 | 81.4 | 0.59 | 0 |
| S041 | 86.9 | 65.23 | 75.1 | 7 | 19.8 | 89.6 | 0.45 | 4 |
| S042 | 90.37 | 77.84 | 86.1 | 10 | 38.3 | 88.9 | 1.08 | 6 |
| S043 | 58.39 | 28.56 | 48.9 | 5 | 78.5 | 94.8 | 1.14 | 4 |
| S044 | 78.06 | 30.74 | 39.4 | 9 | 78.5 | 96.2 | 0.64 | 7 |
| S045 | 84.13 | 68.1 | 80.9 | 17 | 35.1 | 89.1 | 0.58 | 9 |
| S046 | 59.41 | 46.75 | 78.7 | 4 | 43.8 | 93.6 | 0.5 | 3 |
| S047 | 72.12 | 62.47 | 86.6 | 3 | 7.5 | 74.6 | 0.51 | 0 |
| S048 | 66.96 | 52.74 | 78.8 | 6 | 28.9 | 84.7 | 1.31 | 3 |
| S049 | 73.8 | 44.35 | 60.1 | 5 | 24.4 | 92.5 | 0.26 | 3 |
| S050 | 80.62 | 68.38 | 84.8 | 12 | 31.6 | 85.5 | 0.41 | 6 |
| S051 | 75.69 | 58.54 | 77.4 | 8 | 17.4 | 87.4 | 1.02 | 3 |
| S052 | 83.11 | 65.98 | 79.4 | 7 | 38.8 | 89.0 | 0.51 | 2 |
| S053 | 53.31 | 40.04 | 75.1 | 2 | 26.4 | 93.3 | 0.31 | 1 |
| S054 | 65.54 | 22.5 | 34.3 | 5 | 26.0 | 92.0 | 0.46 | 3 |
| S055 | 72.4 | 47.19 | 65.2 | 25 | 27.7 | 89.6 | 0.76 | 14 |
| S056 | 57.01 | 2.03 | 3.6 | 1 | 75.1 | 100.0 | 0.25 | 1 |
| S057 | 72.02 | 47.64 | 66.1 | 18 | 25.6 | 90.9 | 0.51 | 13 |
| S058 | 64.17 | 4.52 | 7.0 | 0 | — | — | — | 0 |
| S059 | 74.01 | 42.68 | 57.7 | 25 | 32.4 | 89.6 | 0.53 | 17 |
| S060 | 63.07 | 5.12 | 8.1 | 0 | — | — | — | 0 |
| S061 | 81.87 | 63.09 | 77.1 | 18 | 42.4 | 94.4 | 0.86 | 12 |
| S062 | 76.77 | 53.32 | 69.5 | 7 | 33.8 | 85.8 | 0.54 | 4 |
| S063 | 85.45 | 6.81 | 8.0 | 2 | 36.6 | 88.4 | 0.58 | 1 |
| S064 | 82.83 | 2.19 | 2.6 | 0 | — | — | — | 0 |
| S065 | 66.68 | 9.6 | 14.4 | 4 | 56.8 | 97.2 | 0.8 | 3 |
| S066 | 73.97 | 38.66 | 52.3 | 5 | 42.6 | 91.4 | 0.66 | 4 |
| S067 | 95.2 | 82.55 | 86.7 | 8 | 29.6 | 89.7 | 0.44 | 5 |
| S068 | 93.68 | 37.04 | 39.5 | 26 | 103.5 | 95.5 | 0.36 | 22 |
| S069 | 82.03 | 6.87 | 8.4 | 1 | 37.9 | 99.9 | 0.01 | 1 |
| S070 | 65.01 | 53.57 | 82.4 | 9 | 30.1 | 94.1 | 0.51 | 7 |
| S071 | 82.72 | 48.05 | 58.1 | 11 | 40.4 | 90.6 | 0.63 | 7 |
| S072 | 69.9 | 50.92 | 72.9 | 6 | 39.7 | 93.1 | 0.82 | 5 |
| S073 | 81.1 | 39.45 | 48.6 | 9 | 34.2 | 88.5 | 0.21 | 5 |
| S074 | 82.34 | 61.26 | 74.4 | 16 | 33.3 | 92.5 | 0.66 | 13 |
| S075 | 96.96 | 81.97 | 84.5 | 21 | 21.7 | 86.7 | 1.04 | 9 |
| S076 | 88.36 | 72.11 | 81.6 | 26 | 20.5 | 85.6 | 0.59 | 12 |
| S077 | 84.58 | 26.47 | 31.3 | 5 | 56.4 | 98.0 | 0.11 | 5 |
| S078 | 76.74 | 55.26 | 72.0 | 6 | 36.7 | 92.6 | 0.17 | 4 |
| S079 | 81.69 | 5.26 | 6.4 | 1 | 89.0 | 99.9 | 0.1 | 1 |
| S080 | 79.51 | 57.97 | 72.9 | 20 | 40.9 | 92.2 | 0.41 | 14 |
| S081 | 88.9 | 64.44 | 72.5 | 21 | 36.8 | 89.0 | 0.45 | 10 |
| S082 | 93.41 | 49.73 | 53.2 | 9 | 17.5 | 84.7 | 1.04 | 5 |
| S083 | 81.97 | 64.76 | 79.0 | 13 | 19.2 | 87.3 | 0.91 | 7 |
| S084 | 80.16 | 66.22 | 82.6 | 20 | 33.6 | 87.1 | 0.84 | 11 |
| S085 | 73.08 | 15.75 | 21.5 | 0 | — | — | — | 0 |
| S086 | 89.19 | 76.36 | 85.6 | 20 | 26.5 | 88.4 | 1.07 | 12 |
| S087 | 81.57 | 71.02 | 87.1 | 16 | 41.6 | 83.4 | 0.59 | 7 |
| S088 | 82.44 | 66.46 | 80.6 | 28 | 27.8 | 87.4 | 1.25 | 13 |
| S089 | 0.34 | 0.27 | 79.9 | 0 | — | — | — | 0 |
| S090 | 85.35 | 3.26 | 3.8 | 0 | — | — | — | 0 |
| S091 | 87.0 | 46.8 | 53.8 | 6 | 15.1 | 87.7 | 0.78 | 4 |
| S092 | 94.03 | 62.7 | 66.7 | 10 | 29.0 | 90.6 | 0.5 | 8 |
| S093 | 78.6 | 14.19 | 18.1 | 5 | 60.0 | 97.1 | 0.44 | 5 |
| S094 | 106.22 | 26.45 | 24.9 | 12 | 100.6 | 94.4 | 0.39 | 9 |
| S095 | 62.79 | 36.68 | 58.4 | 13 | 104.4 | 95.9 | 0.27 | 11 |
| S096 | 84.81 | 73.31 | 86.4 | 30 | 25.8 | 85.3 | 0.69 | 12 |
| S097 | 85.71 | 29.68 | 34.6 | 3 | 210.6 | 99.5 | 0.17 | 3 |
| S098 | 87.47 | 61.41 | 70.2 | 5 | 30.3 | 90.6 | 0.25 | 3 |
| S099 | 96.93 | 72.51 | 74.8 | 23 | 35.9 | 87.4 | 0.58 | 12 |
| S100 | 73.15 | 58.94 | 80.6 | 18 | 25.3 | 86.5 | 0.37 | 10 |
| S101 | 82.86 | 41.21 | 49.7 | 5 | 25.2 | 83.5 | 0.59 | 1 |
| S102 | 77.07 | 5.73 | 7.4 | 4 | 72.8 | 99.9 | 0.06 | 4 |
| S103 | 67.26 | 58.26 | 86.6 | 5 | 29.1 | 93.4 | 1.03 | 4 |
| S104 | 91.28 | 61.33 | 67.2 | 15 | 23.0 | 90.0 | 0.78 | 9 |
| S105 | 90.85 | 35.75 | 39.3 | 5 | 31.4 | 93.0 | 0.45 | 4 |
| S106 | 88.14 | 47.4 | 53.8 | 10 | 39.4 | 88.1 | 0.76 | 5 |
| S107 | 96.03 | 78.95 | 82.2 | 23 | 26.4 | 87.5 | 0.77 | 13 |
| S108 | 99.49 | 86.13 | 86.6 | 38 | 32.2 | 85.4 | 0.76 | 18 |
| S109 | 95.19 | 77.11 | 81.0 | 27 | 27.5 | 90.4 | 1.05 | 18 |
| S110 | 108.96 | 92.42 | 84.8 | 30 | 25.3 | 88.3 | 0.64 | 18 |
| S111 | 106.19 | 91.86 | 86.5 | 29 | 30.4 | 91.5 | 0.59 | 20 |

## Supplementary Figures


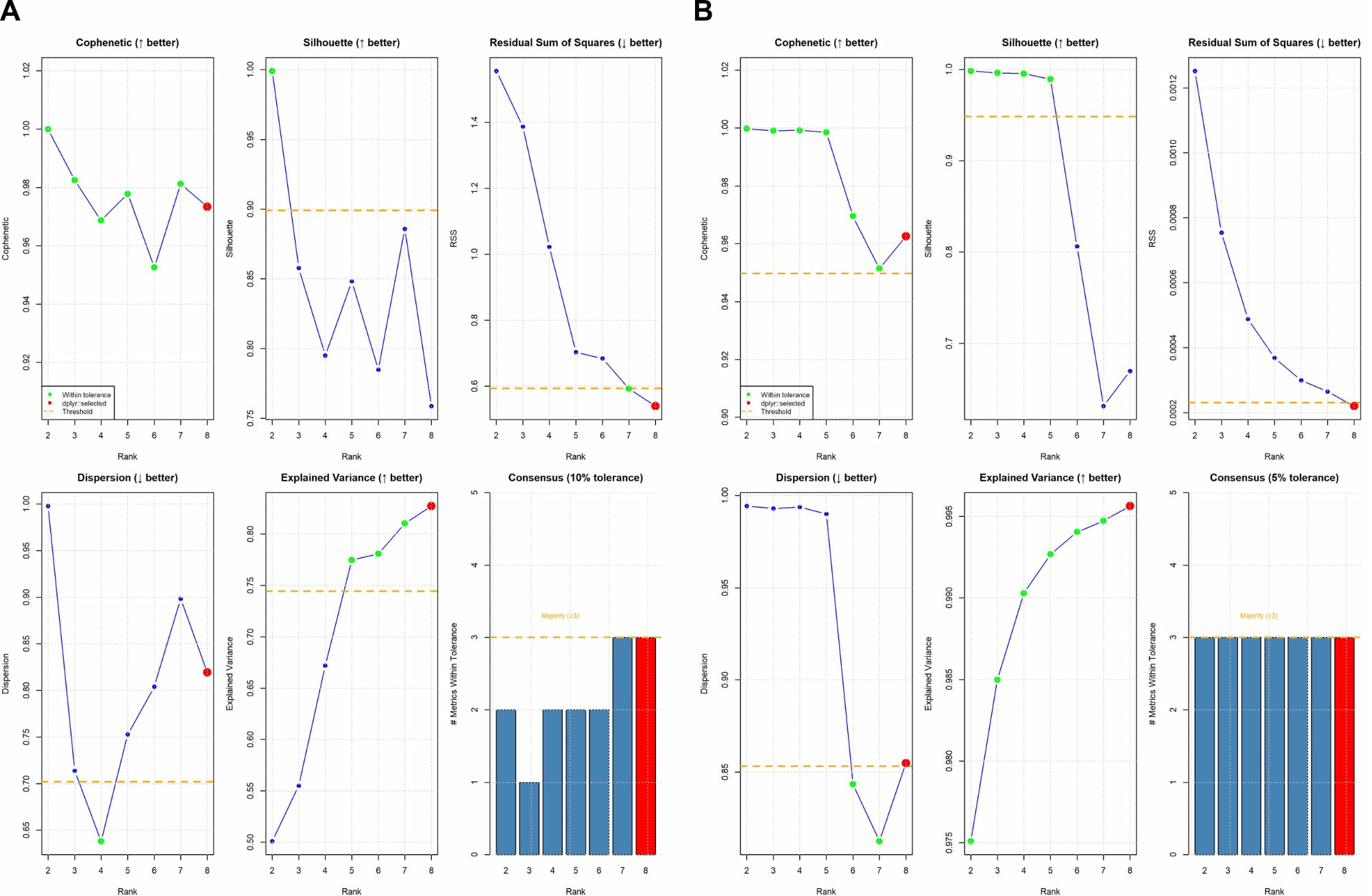


**Supplementary Figure 1. NMF rank selection for taxonomic and functional enterosignatures.** (A) Taxonomic enterosignatures (T-ES) derived from the MAG abundance matrix. (B) Functional enterosignatures (F-ES) derived from the KO abundance matrix. For each data layer, five consensus metrics were evaluated across ranks k = 2–8: cophenetic correlation coefficient (↑ better), silhouette width (↑ better), residual sum of squares (RSS; ↓ better), dispersion coefficient (↓ better), and explained variance (↑ better). Green points indicate ranks within the tolerance threshold (dashed orange line) for each metric; the red point marks the selected rank. The consensus barplot (bottom right of each panel) shows the number of metrics within tolerance at each rank. For the MAG matrix, ranks k = 4, 7, and 8 each had 3 of 5 metrics within tolerance; k = 8 was selected to maximize ecological resolution while remaining within the consensus-supported range. For the KO matrix, k = 5 achieved the highest consensus (5 of 5 metrics within tolerance) and was selected. NMF was run using the Brunet algorithm with 200 iterations and 30 runs per rank.


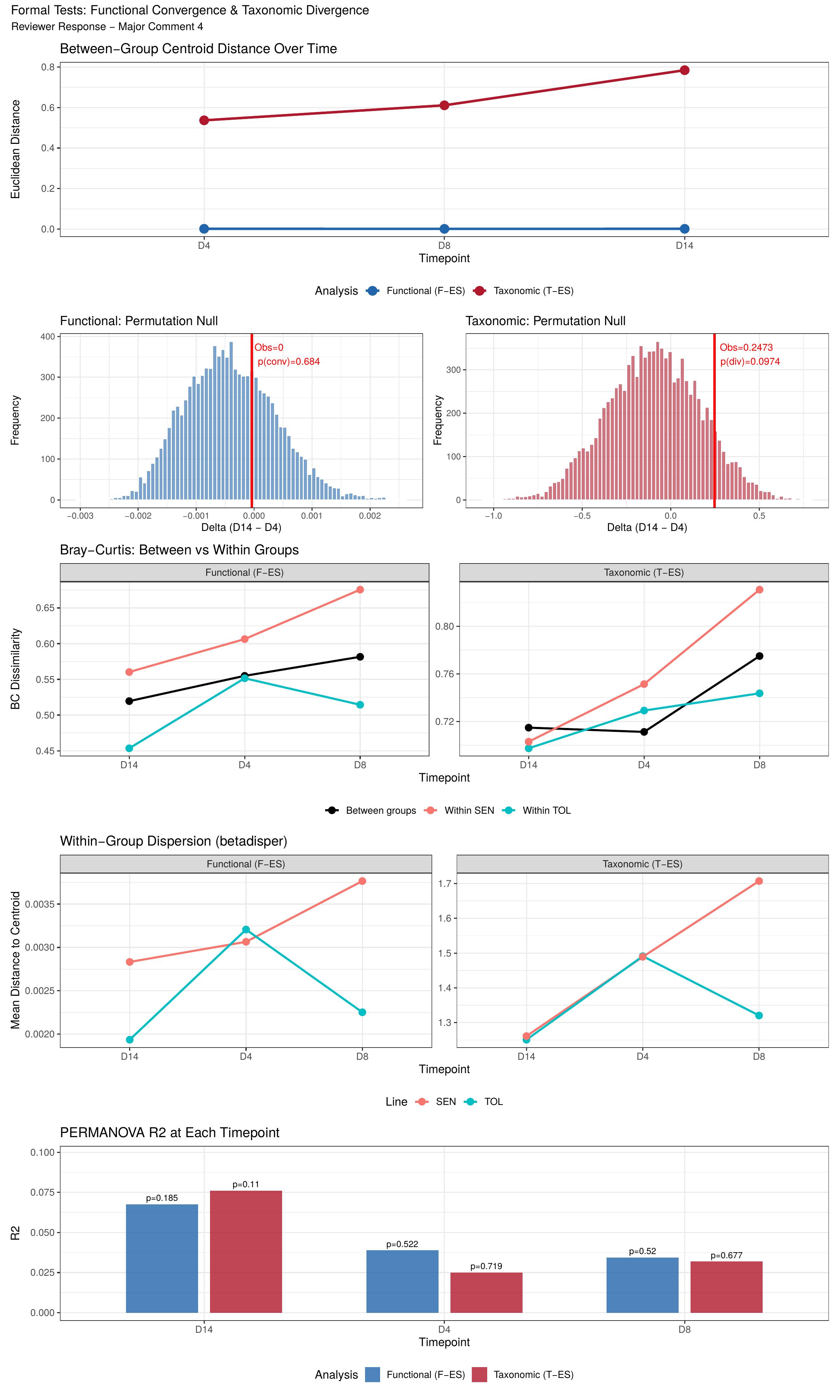


**Supplementary Figure 2. Formal convergence and divergence testing of functional and taxonomic enterosignatures between genetic lines.** Between-group (TOL vs. SEN) centroid distances were computed in NMF W-matrix space at each timepoint (D4, D8, D14) for both functional (F-ES, k = 5) and taxonomic (T-ES, k = 8) enterosignatures. (A) Centroid distance trajectories showing the change in between-group separation over time. Functional ES centroid distances remained near zero across all timepoints, indicating maintained equivalence between genetic lines. Taxonomic ES centroid distances increased from D4 to D14. (B–C) Permutation null distributions (9,999 permutations, group labels shuffled within timepoints) for the change in centroid distance (Δ = dist(D14) – dist(D4)). (B) Functional ES: Δ ≈ 0.00, p(convergence) = 0.684, indicating no directional change. (C) Taxonomic ES: Δ = 0.247, p(divergence) = 0.097, indicating a trend toward divergence that approached but did not reach conventional significance. (D) Bray–Curtis dissimilarities decomposed into between-group and within-group components at each timepoint, showing stable functional dissimilarity and increasing taxonomic between-group distance. (E) Betadisper analysis of within-group multivariate dispersion for each genetic line across timepoints. (F) PERMANOVA R² values for genetic line at each timepoint, showing negligible variance explained in functional space (all R² < 0.07, all p > 0.18) and increasing variance explained in taxonomic space (D14 R² = 0.076, p = 0.110).


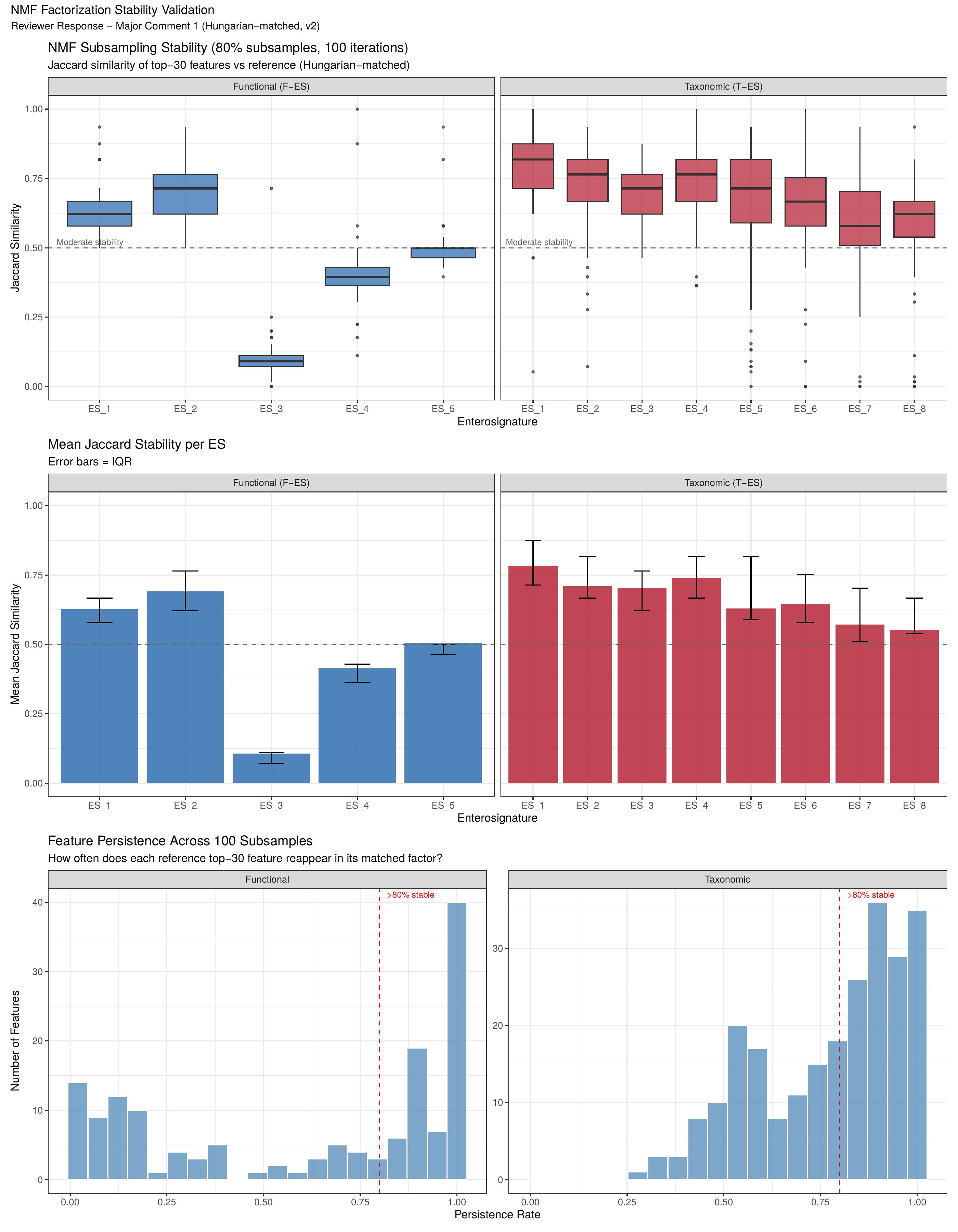


**Supplementary Figure 3. Subsampling stability validation of NMF enterosignature solutions.** NMF was refitted on 100 random 80% subsamples of the original sample set at the established ranks (k = 8 for MAGs, k = 5 for KO). For each subsample iteration, the top features (based on H-matrix loadings) of each recovered factor were compared to those of the reference (full-dataset) solution using Jaccard similarity, with optimal factor-to-reference matching determined by the Hungarian algorithm. (A) Boxplots of Jaccard similarity distributions for taxonomic enterosignatures (T-ES 1–8). T-ES showed strong overall stability (mean Jaccard = 0.67): four signatures achieved strong stability (T-ES 1 = 0.78, T-ES 4 = 0.74, T-ES 2 = 0.71, T-ES 3 = 0.70), two showed moderate-to-strong stability (T-ES 6 = 0.65, T-ES 5 = 0.63), and two showed moderate stability (T-ES 7 = 0.57, T-ES 8 = 0.55). Six of eight taxonomic signatures achieved mean Jaccard ≥ 0.60. (B) Boxplots of Jaccard similarity distributions for functional enterosignatures (F-ES 1–5). F-ES stability was more variable (mean Jaccard = 0.47): F-ES 2 (0.69) and F-ES 1 (0.63) showed strong to moderate-strong stability, F-ES 5 (0.50) showed moderate stability, F-ES 4 (0.41) showed moderate-weak stability, and F-ES 3 (0.11) was unstable. The instability of F-ES 3 reflects its low prevalence in the reference solution (dominant in only 3 of 74 samples), making it difficult to recover consistently from 80% subsamples. Feature persistence rates at the ≥80% threshold were 56.2% for taxonomic and 49.3% for functional enterosignatures.


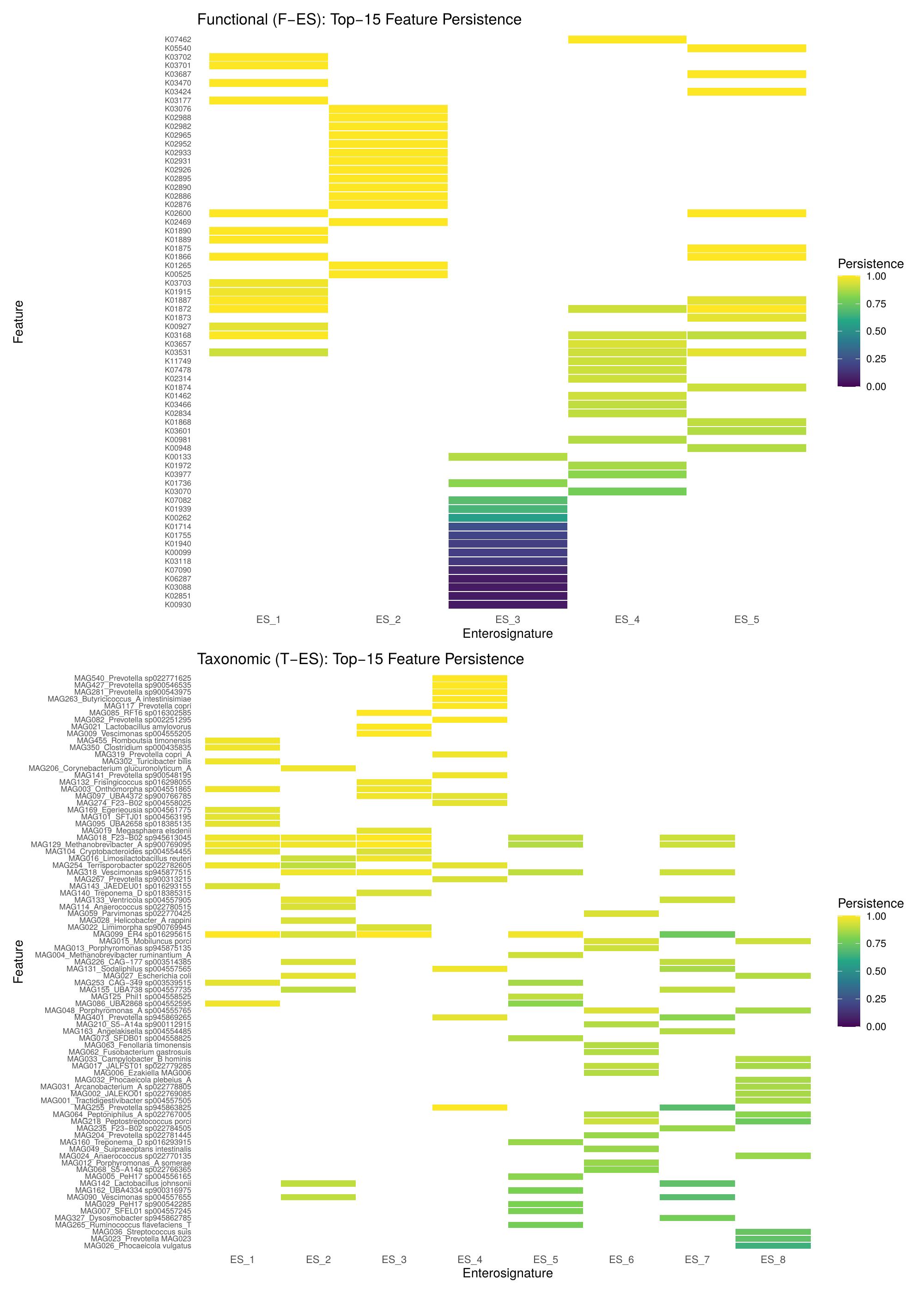


**Supplementary Figure 4. Feature persistence across subsampling iterations for taxonomic and functional enterosignatures.** Heatmaps showing the proportion of 100 subsampling iterations in which each top feature (rows) was recovered within the matched enterosignature. Features are ordered by persistence rate within each signature. (A) Taxonomic enterosignature feature persistence (T-ES 1–8). Most top MAG features showed high persistence (≥80% of iterations), confirming that the defining taxa of each T-ES are robust to sample perturbation. Core features of the most stable signatures (T-ES 1, 2, 3, 4) were recovered in >90% of iterations. (B) Functional enterosignature feature persistence (F-ES 1–5). KO feature persistence was more heterogeneous, reflecting the larger feature space and the inherent redundancy of functional annotations. F-ES 2 and F-ES 1 showed the highest feature persistence, consistent with their strong Jaccard similarity scores (Supplementary Figure 3). F-ES 3 showed minimal feature persistence, consistent with its instability noted above. Color intensity reflects the proportion of iterations (0–1.0) in which each feature was among the top features of the matched signature.
